# Selective isolation of Arctic marine actinobacteria and a down-scaled fermentation and extraction strategy for identifying bioactive compounds

**DOI:** 10.3389/fmicb.2022.1005625

**Published:** 2022-11-21

**Authors:** Yannik K. Schneider, Ole C. Hagestad, Chun Li, Espen H. Hansen, Jeanette H. Andersen

**Affiliations:** ^1^Marbio, Faculty for Fisheries, Biosciences and Economy, UiT—The Arctic University of Norway, Tromsø, Norway; ^2^Marbank, Institute of Marine Research, Tromsø, Norway

**Keywords:** actinobacteria, isolation, natural products, bioprospecting, marine, Arctic, antibiotics

## Abstract

Actinobacteria are among the most prolific producers of bioactive secondary metabolites. In order to collect Arctic marine bacteria for the discovery of new bioactive metabolites, actinobacteria were selectively isolated during a research cruise in the Greenland Sea, Norwegian Sea and the Barents Sea. In the frame of the isolation campaign, it was investigated how different sample treatments, isolation media and sample-sources, such as animals and sediments, affected the yield of actinobacterial isolates to aid further isolation campaigns. Special attention was given to sediments, where we expected spores of spore forming bacteria to enrich. Beside actinobacteria a high share of bacilli was obtained which was not desired. An experimental protocol for down-scaled cultivation and extraction was tested and compared with an established low-throughput cultivation and extraction protocol. The heat-shock method proved suitable to enrich spore-, or endospore forming bacteria such as bacilli. Finally, a group bioactive compounds could be tentatively identified using UHPLC–MS/MS analysis of the active fractions.

## Introduction

Among the realm of Prokaryota, the actinobacteria are representing a phylum that is rather different from the otherwise predominantly monocellular bacteria with commonly circular genomes. Actinobacteria are Gram positive, spore forming bacteria with high CG content and some of them, in particular the genus *Streptomyces*, show filamentous growth and some have linear genomes (Nett et al., 2009; Barka et al., 2016). For natural product chemists, the most interesting property of the actinobacteria is their high biosynthetic potential. Actinobacteria, and in particular the genus *Streptomyces*, were the source of a significant share of the natural products that have been developed into drugs (János, 2005; Jose and Jha, 2016). Many of the currently available antibiotics origin from *Streptomyces*, but also bioactive molecules belonging to other drug-classes such as the anti-cancer drug actinomycin (Sakula and Sakula, 1988), the immunosuppressant rapamycin (Vezina et al., 1975; Arriola Apelo and Lamming, 2016) and the pancreatic lipase inhibitor lipstatin (Hochuli et al., 1987) originated from actinobacteria. We have previously outlined a strategy to increase the hit rate when screening bacterial isolates for bioactivities and novel compounds, mainly based on the selection of promising genera (Schneider, 2021). The strategy is based on the observation that the biosynthetic potential of bacteria is not equally distributed as some taxonomic groups stand out. The genome size of bacteria appears to be an good indicator for the biosynthetic potential, since polyketide-synthetases (PKS) and non-ribosomal-peptide synthetases (NRPS) are rare or absent in genomes smaller than 3 Mb (Donadio et al., 2007). The number of biosynthetic gene clusters (BGCs) in *Streptomyces* is also positively correlated to genome size, which is another link between genome size and biosynthetic potential (Belknap et al., 2020). A further indicator for biosynthetic potential seems to be the complex morphology and life-cycle of the respective bacteria (Schneider, 2021). The taxa that seem to be most prolific producers of bioactive secondary metabolites are actinobacteria, cyanobacteria, myxobacteria and bacilli (Schneider, 2021). Apparently, Gram positive bacteria play a dominant role as producer of bioactive natural products, in particular antibiotics. Gupta (2011) suggests that the cell-wall of Gram negative bacteria evolved as a response to the selection pressure of antibiotics, which are mostly produced by the evolutionary older Gram positive bacteria. This would give an evolutional explanation to the decreased antibiotic sensitivity of Gram negative bacteria as well as the role of the Gram positive bacteria as main antibiotic producers (Schinke et al., 2017). Given the past and current output of bioactive natural products from actinobacteria (Genilloud, 2017), we decided to focus on the isolation of actinobacteria from field samples to increase the hit rate in our screening campaigns for antibacterial, antifungal, anti-cancer, anti-inflammatory and anti-diabetic activities of bacterial extracts.

Rare actinobacteria have also become subject to bioprospecting efforts after the initial focus on *Streptomyces* (Bredholdt et al., 2007; Dhakal et al., 2017; Bundale et al., 2019; Subramani and Sipkema, 2019; Amin et al., 2020). The term of “rare actinobacteria” describes genera of actinobacteria for which the isolation frequency using conventional methods is lower than for *Streptomyces* (Benhadj et al., 2019). From the 1950s to the 1970s, the bulk of antibiotics were isolated from *Streptomyces*, but the rare actinobacteria have drawn more attention from the 1980s onwards with a share of antibiotics increasing from ~5% to ~30% during the 1980s and being ~10% by 2012 (János, 2012). New antibiotics are urgently needed (Spížek et al., 2010; Khan and Khan, 2016), and actinobacteria have so far delivered 90% of the commercial antibiotics (Jose and Jha, 2016). There are 50 taxa of rare actinomycetes reported to produce about 2,500 bioactive compounds (Kurtböke, 2012) and for the genus *Streptomyces* it was estimated by 2001 that only 3% of the compounds have been discovered and about 150,000 bioactive compounds await discovery (Watve et al., 2001).

In 2017 Schinke et al. reviewed the antibacterial compounds (setting an activity threshold) from marine bacteria discovered between 2010 and 2015 (Schinke et al., 2017). Actinobacteria and bacilli where the most prolific producers with 27 and 12 new compounds, respectively. They also indicated the origin of the bacteria, such as sediment, sponges, algae etc. and the sediment contributed ~ ¾ of the actinobacteria and > ½ of the bacilli producing anti-bacterial compounds. We were wondering if this is due to a sampling bias, since sediment sampling is easy to facilitate, or if it is due to enrichment of bacterial spores in sediment. Actinobacteria form spores and bacilli are capable of endospore formation, we hypothesized that spores, at least to some extent, sediment from the water column to the seabed and endure there, which would make seabed-sediment a good source to isolate spore forming bacteria from.

We aimed for isolating marine actinobacteria on a research cruise in the Greenland Sea, Barents Sea and the Norwegian Sea. We were not able to find a direct experimental comparison of the efficacy of the methods, especially for marine actinomycetes for our sampling area. We decided to focus our isolation campaign on isolation of actinobacteria and on comparing the suitability of isolation media, sample preparation and sample-origin (mainly animals and sediments), in order to optimize future isolation strategies to increase the yield of actinobacterial isolates for the discovery of new bioactive compounds.

## Materials and methods

Filtrated seawater (FSW) for the respective experiments was produced from the seawater supply of the Norwegian College of Fishery Science (Tromsø, Norway) through a Millidisk® 40 cartridge with Durapore® 0.22 μm filter membrane (Millipore, Burlington, MA, USA). Purified water (pH_2_O) was produced by the in-house MilliQ system. Equipment, consumables, chemicals and media were autoclaved at 121°C for 30 min under increased pressure (MLS-37812, Panasonic, Kadoma, Japan).

### Sample collection

The sediment-and animal samples for isolation of bacteria were collected during a research cruise with the Norwegian research vessel Kronprins Haakon. The animal samples were collected by bottom-trawling and sediments from the seabed were collected using a box corer. A 50 × 50 × *ca.* 50 cm sediment cube was sampled and recovered with the seabed-surface still intact, pictures from the sediment sampling and trawling equipment are given in the Supplementary information (Supplementary Figure 1). Sediment samples were also collected by hand from the intertidal zone at the island Bjørnøya together with soil samples from the vegetative zone adjoining the beach.

### Sample preparations

Samples for isolation of bacteria were taken with a sterile spatula from the surface of the seabed that was recovered by the box corer. From each sediment core one sample from the surface and one sample from-10 cm under the seabed’s surface were taken. Approximately 100 μl of sediment were dissolved in 900 μl autoclaved FSW.

Samples from animals, macroalgae and driftwood were processed as follows. The sample was washed under autoclaved filtrated seawater, a small piece from the organism, if possible, the inside/ no surface tissue, was transferred to an autoclaved 1.5 ml reaction tube, and a plastic pestle fitting the geometry of the tube was used to grind the sample, if necessary autoclaved filtrated seawater was added to enable to grind the animal into a semi-liquid homogenate. Preparation of the homogenates and sample-cuts was done using sterile equipment and a Bunsen-burner to create a sterile air-cone to avoid contamination.

### Preparation of media used for isolation and cultivation of actinobacteria

M1 media: 10.0 g starch, 4.0 g yeast extract, 2.0 g peptone, 1.0 l filtrated seawater (Webster et al., 2001; Abdelmohsen et al., 2010)

R2A media: 0.5 g yeast extract, 0.5 g peptone, 0.5 g casaminoacid, 0.5 g glucose, 0.5 g starch, 0.3 g K_2_HPO_4_, 0.05 g MgSO_4_ 7 × H_2_O, 0.3 g Na-pyruvate, 1.0 l filtrated seawater (Reasoner and Geldreich, 1985; Massa et al., 1998; Ballav et al., 2015).

AiA media: 1.0 g peptone, 0.1 g asparagine, 4.0 g Na-propionate, 0.5 g K_2_HPO_4_, 0.1 g MgSO_4_, 1.0 mg FeSO_4_ 7 × H_2_O, 1 ml glycerol, 1.0 l filtrated seawater. (Webster et al., 2001; Abdelmohsen et al., 2010).

FMAP Media: 15.0 g Difco marine broth, 5.0 g peptone, 300 ml filtrated seawater, 700 ml pH_2_O.

ISP2-Media with seawater: 4.0 g glucose, 4.0 g yeast extract, 10.0 g malt extract, 300 ml filtrated seawater, 700 ml pH_2_O + 0.2% (v/v) trace element solution.

Mueller-Hilton Broth: Difco 275,730, 21 g dissolved in 1 l of pH_2_O, approx. Composition according to manufacturer: 2.0 g beef extract powder, 17.5 g acid digest of casein, 1.5 g soluble starch per L.

Trace element solution: 10% MgSO_4_ 7 × H_2_O, 0.01% FeSO_4_ 7 × H_2_O, 0.01% ZnSO_4_ 7 × H_2_O, 0.01% CuSO_4_ 5 × H_2_O, 0.01% CoCl_2_ 6 × H_2_O all (w/v) dissolved in pH_2_O.

For preparation of solid media to be poured in petri-dishes 20 g agar powder was added per liter of medium prior to autoclavation.

After the preparation the media were autoclaved at 121°C for 30 min. For gelatinization, 2.0% (w/v) of agar powder were added before autoclavation and the gelatinized media was poured in petri-dishes under a laminar flow. Petri-dishes with gelatinized media were stored at 4°C in darkness until use.

### Addition of antibiotics and fungicide to the isolation media

The three isolation media (AiA, M1 & R2A) were supplemented with antibiotics after autoclavation and cooling to ~45°C. A stock solution of cycloheximide was prepared by dissolving it in EtOH to a final concentration of 10 mg/ml. Naldixic acid stock solution was prepared by dissolving it in 0.3 M NaOH to a final concentration of 30 mg/ml. The naldixic acid stock solution was sterile filtrated using a 25 ml disposable syringe and a 25 μm sterile filter into an autoclaved glass bottle under a laminar flow. For each isolation media, 5.0 ml of the 10 mg/ml cycloheximide stock solution were added and 1.0 ml of 30 mg/ml naldixic acid stock solution to reach a final concentration of 50 mg/l cycloheximide and 30 mg/l naldixic acid within the isolation agar. The media was poured into petri-dishes directly after the antibiotics were added.

### Isolation of bacteria and plating strategy

**Figure 1:**
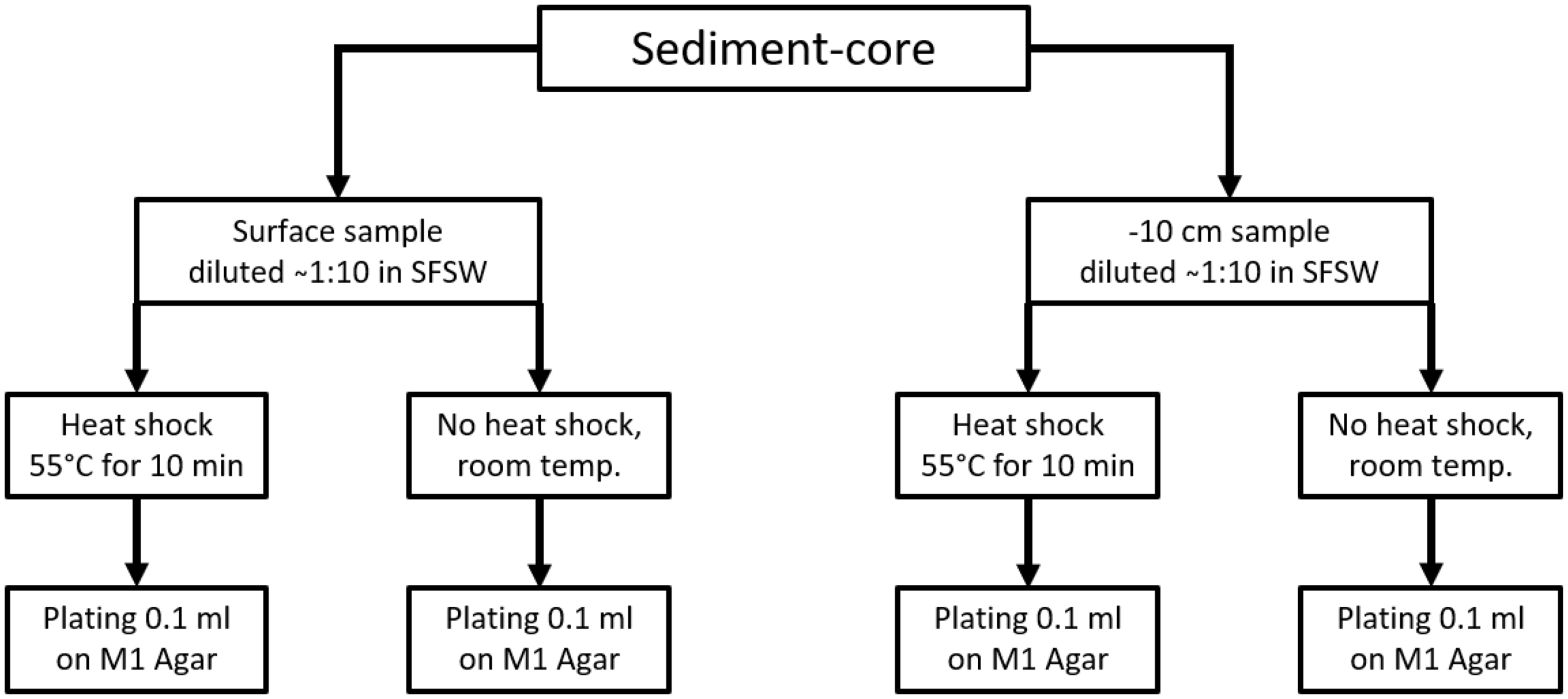
Sample process and plating scheme for the sediment samples. From each sediment core one surface sample and one sample from-10 cm under surface. Each sample was processed once using the heat-shock pre-treatment and once without heat shock. All sediment samples were plated on M1 isolation agar, the same scheme was applied for the sediments retrieved from Bjørnøya.

**Figure 2:**
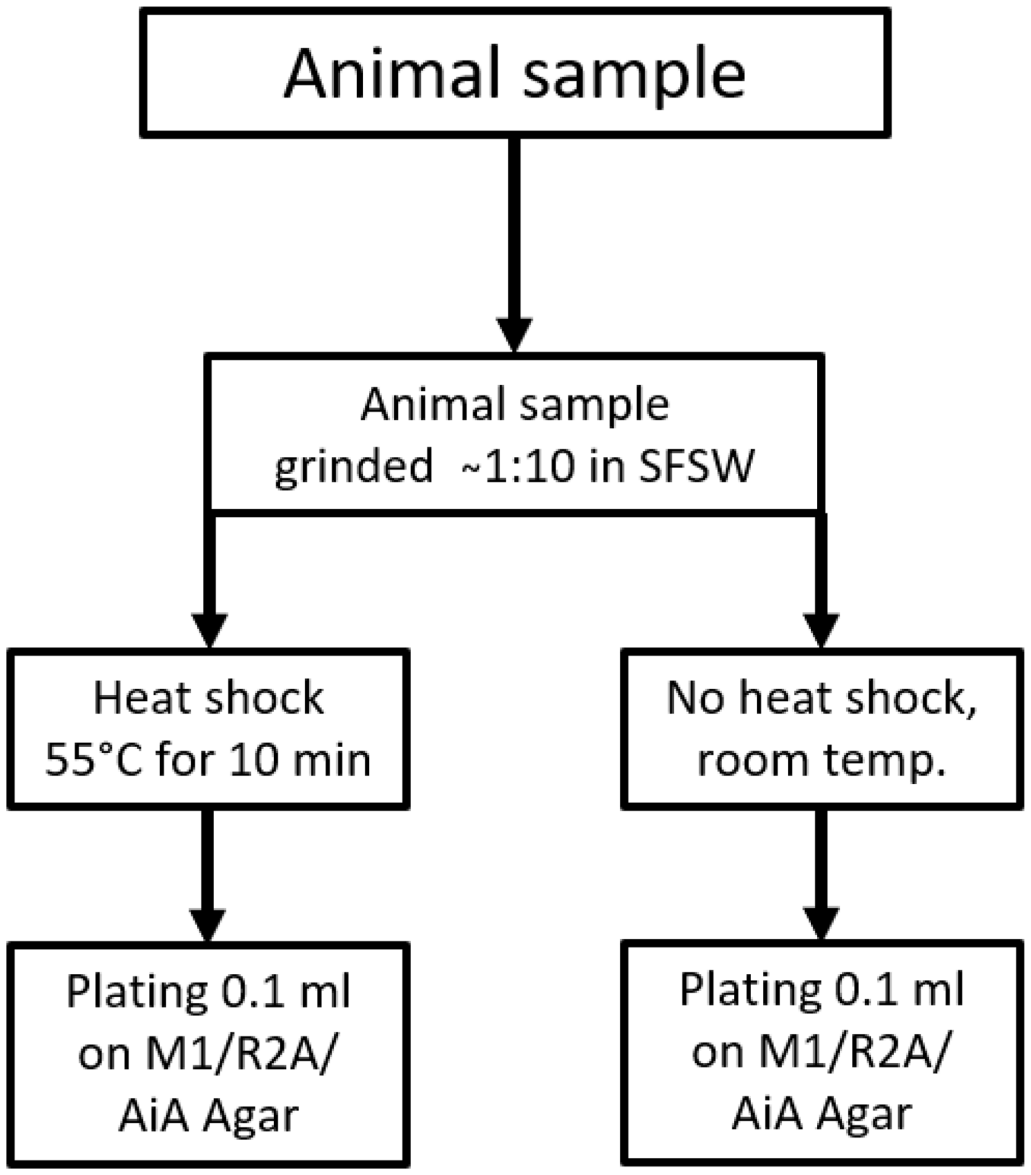
Sample treatment and plating scheme for animal and “other” samples, animal samples were plated on M1, AiA & R2A isolation agar.

From each sediment core two samples were taken, one from the surface and one from 10 cm below the surface, while only one sample was taken from animal samples or other solid biomass samples. For plating, two aliquots of each sample were prepared. One aliquot was heated to 55°C for 10 min using an electrically heated thermo-block and 1.5 ml reaction tubes, and this heat treatment was meant to increase the ratio of spore-forming bacteria among the isolated species. The other aliquot was kept at ambient temperature. The samples were plated using sterile equipment and a Bunsen-burner to create a sterile air-flow to avoid contamination. For each isolation-agar-plate 100 μl of homogenate were plated out using a single-use Drigalski-spatula, the plates were closed using Parafilm® and incubated at 20°C for 2–3 months. The plating strategy is shown in Figure 1 for sediment samples and Figure 2 for all “non sediment” samples. For each animal sample one aliquot of homogenate was exposed to heat treatment (55°C for 10 min) the other one was kept at ambient temperature, before both were plated on isolation-agar. The sediment samples were processed similarly, one aliquot was exposed to heat and one was kept at ambient temperature before plating. M1-isolation-media was used for all sediment samples. For the other samples M1-, R2A-and AiA-isolation media were used. Samples of each homogenate were mixed with autoclaved filtrated seawater containing 30% glycerol to a final concentration of 15% glycerol and cryo-conserved at-80°C as retention sample for potential further investigation. The processing and plating of the samples on isolation agar was done in the laboratory facilities on board the research vessel and were finished less than 5 h after sampling.

When colonies appeared on the plates with isolation media, the colonies were visually inspected. After inspection one colony of every morphological group of colonies that could be observed on an isolation plate was picked and transferred on FMAP agar. If not axenic colonies were picked and transferred another time on FMAP agar until isolates were obtained. For the phylogenetic identification of the bacterial strains, one colony was picked using a 100 μl pipet tip and the bacterial biomass was dissolved in 100 μl of autoclaved pH_2_O for further identification *via* colony-PCR.

Freeze stocks of the isolated strains were prepared by inoculating 5.0 ml of FMAP liquid medium with a colony of the respective isolate and incubation at 20°C until sufficient turbidity was reached. For cryopreservation 0.5 ml of the respective culture were transferred into a cryo-tube and mixed with 1.0 ml FMAP liquid medium containing 30% (v/v) glycerol. The cryo-samples were stored at-80°C.

### Colony PCR and 16S-rRNA sequencing

The phylogenetic analysis of the bacterial isolates was done by sequencing of the 16S rRNA gene through colony PCR and Sanger sequencing. The primer set used for amplification of the gene was the 27F primer (forward primer; 5’-AGAGTTTGATCMTGGCTCAG) and the 1429R primer (reverse primer; 5’-TACCTTGTTACGACTT), both from Sigma. Prior to the amplification PCR, the bacterial samples were vortexed and diluted 1:100 and 1:1000 in UltraPureWater (BioChrom GmbH, Berlin, Germany). For PCR, 1 μl of the diluted bacterial sample was combined in a 25-μL PCR reaction, together with 12.5 μl DreamTaq Green PCR Master Mix (2×; Thermo Scientific, Vilnius, Lithuania), 10.5 μl ultrapure water, and 0.5 μl of the forward and reverse primers (10 μM) mentioned above. The amplification was done using a Mastercycler ep gradient S (Eppendorf AG, Hamburg, Germany) with the following program: 95°C initial denaturation for 3 min, followed by 35 cycles of 95°C for 30 s, 47°C for 30 s, and 72°C for 1 min. Final extension was 72°C for 10 min. The success and purity of the PCR reaction was analyzed on a 1.0% agarose gel (Ultrapure™ Agarose, Invitrogen, Paisley, UK) with Gel-Red® Nucleic Acid Gel Stain (Biotium, Fremont, CA, USA), and the results were documented using a Syngene Bioimaging system (Syngene, Cambridge, UK). Successfully amplified samples were purified by the A’SAP PCR clean up kit (ArcticZymes, Tromsø, Norway). The purified PCR product was used for sequencing PCR, using 1 μl PCR product, 2 μl BigDye™ 3.1 (Applied Biosystems, Foster City, CA, USA), 2 μl 5 × sequencing buffer (Applied Biosystems, Foster City, CA, USA), 4 μl of UltraPure water, and 1 μl of primer (1 μM of 27F primer or 1429R primer). The program for the sequencing PCR was as follows: 96°C initial denaturation for 1 min, followed by 30 cycles of 96°C for 10 s, 47°C for 5 s, and 60°C for 2 min. The PCR product was sequenced at the University Hospital of North Norway (Tromsø, Norway) using a Applied Biosystems 3130xl Genetic Analyser (Life Technologies/Applied Biosystems, Waltham, MA, USA).

### Isolate identification

Each 16s sequence was submitted to GenBank for a BLAST search, limiting hits to type sequences and excluding uncultured, metagenomic, and environmental samples (NCBI, 2015; Raja et al., 2017). The hits for each sequence were downloaded and aligned. Alignment of the sequences were done in Geneious Prime 2022.1.1,^1^ using MAFFT v.7.450 (Katoh et al., 2002; Katoh and Standley, 2013) with the G-INS-I algorithm and scoring matrix PAM100/k = 2. The alignment was then manually controlled and single nucleotide noninformative sites were removed.

The resulting alignment and model were used for Maximum likelihood and Bayesian tree construction using RAxML v8.2.11 (Stamatakis, 2014) and MrBayes v.3.2.6 (Altekar et al., 2004; Ronquist et al., 2012) in Geneious. RAxML was run with the following settings: Substitution model GTR gamma, rapid bootstrapping and search for best scoring ML tree with 2000 bootstrap replicates. The nexus file with alignments and MrBayes command block can be found in the Supplementary data. The built in Tracer in Geneious was used to assess the convergence of the MrBayes runs (Rambaut et al., 2018). The optimization of proposal mechanisms was done according to Ronquist et al. (2012). The analysis was run until the standard deviation of the split frequency stabilized at a value or dropped below 0.05.

*Chloroflexus* was selected as outgroup because it is the phylum most closely related to actinobacteria based on the phylogenetic tree presented in Hug et al. (2016).

### Cultivation of actinobacteria and bio activity testing in 96-well plates

A 96-well plate with 1.2 ml FMAP-medium per well was inoculated with the 63 isolated actinobacteria, one well was assigned to one isolate and the plate was incubated for 1 week at room temperature. Another two 96-well plates with M-medium were filled with 1.0 ml medium per well and each well was inoculated with 200 μl of the 2 week old cultures. The two duplicate plates were cultivated for 14 days at room temperature and were subsequently extracted/ harvested. For extraction the plates were frozen at −20°C over night and subsequently thawed. From thereon, the plates were treated differently. One plate was centrifuged (4,500 rpm for 15 min using a Heraeus Multifuge X3, Hanau, Germany, equipped with a Rotor 75003606, ThermoFisher, Waltham, MA, USA) to sediment the cells and the supernatants were pipetted into a new 96-deep-well plate and stored at-20°C until they were tested for anti-microbial activity testing. The second plate was dried using a Speed-Vac concentrator for 48 h *in vacuo* at 45°C (Practical comment: Attempts to freeze-dry deep-well plates with about over 0.5–1.0 ml aqueous-solution per well revealed the problem of the ice-cubes within the wells being lifted off due to the generated gas-pressure underneath which pushes the ice up the wells and it eventually falls into other wells). The dry cultures were extracted with 90% methanol (MeOH) aq. for 2 days, shaking at 260 rpm covered with a rubber lid closing every well individually. The plates were centrifuged at 5000 rpm for 10 min, and the MeOH supernatant was transferred to a new deep-well-plate and reduced to dryness *in vacuo* at 45°C by vacuum centrifugation. The extract was then dissolved in 50 μl dimethyl sulfoxide (DMSO), shaken for 2 h and subsequently added 150 μl of pH_2_O to finally dissolve the extract in 200 μl of 25% (v/v) DMSO aq.

Four of the isolated actinobacteria were cultivated and extracted using our conventional extraction protocol employing a poly-benzyl resin. Starter cultures of the isolates T020, T022, T045 and T252 in 50 ml FMAP media were inoculated with the respective freeze-stocks and incubated at 28°C for 2 days. For each isolates two autoclaved 1.0 l Warburg flasks, each containing 500 ml of ISP1 media, were inoculated with 25 ml of starter culture and cultivated at 28°C for 14 days shaking at 100 rpm using a shanking-incubator (Multitron Pro, INFORS HT, Bottmingen, Switzerland). Cultures were extracted as described here (Schneider et al., 2021) and the extracts were fractionated into six fractions using FLASH-liquid chromatography as described here (Schneider et al., 2019).

The extracts were screened for anti-microbial activity against *Staphylococcus aureus* (ATCC 25923), *Escherichia coli* (ATCC 259233), *Enterococcus faecialis* (ATCC 29122), *Pseudomonas aeruginosa* (ATCC 27853), *Streptococcus agalactiae* (ATCC 12386), and Methicillin-resistant *Staphylococcus aureus* (MRSA; ATCC 33591). All isolates were provided by LGC Standards (Teddington, London, UK). *S. aureus*, MRSA, *E. coli*, and *P. aeruginosa* were grown in Muller Hinton broth (275730, Becton). *E. faecalis* and *S. agalactiae* were cultured in brain hearth infusion broth (53286, Sigma). Fresh bacterial colonies were transferred to the respective medium and incubated at 37°C overnight. The bacterial cultures were diluted to a culture density representing the log phase, and 50 μl/well were pipetted into a 96-well microtiter plate (734–2097, Nunclon™, Thermo Scientific, Waltham, MA, USA). The final cell density was 1′500–15′000 colony forming units/well. Extracts were diluted in 2% (v/v) DMSO (Dimethyl sulfoxide) in pH_2_O, and the final assay concentration was 50% of the prepared sample, as 50 μl of sample in DMSO/water were added to 50 μl of bacterial culture. After adding the samples to the plates, they were incubated over night at 37°C and the growth was determined by measuring the optical density at λ = 600 nm (OD600) with a 1420 Multilabel Counter VICTOR3™ (Perkin Elmer, Waltham, MA, USA). A water sample was used as the reference control, growth medium without bacteria as a negative control, and a dilution series of gentamycin (32 to 0.01 μg/ml, A2712, Merck) as the positive control and visually inspected for bacterial growth. The positive control was used as a system suitability test and the results of the antimicrobial assay were only considered valid when the positive control was passed. For the testing of the bacterial supernatant and methanol-extracts only *S. agalactiae* and *P. aeruginosa* were used because of the small quantity of extract obtained from the 96-well plate cultures.

Screening for cytotoxic activity against human cell lines was only done for the resin-extracts due to sterility concerns for the cell-laboratory (spores from actinobacteria in supernatant). The cell lines tested were MV411 (acute lymphoblastic leukemia, ATCC CRL-1582™) MOLT-4 (Acute lymphoblastic leukemia, ATCC CRL-1582™) and MRC-5 (lung-fibroblasts, ATCC CCL-171™). The cells were cultured and assayed in Roswell Park Memorial Institute medium (RPMI-16040, FG1383, Merck) containing 10% (v/v) fetal bovine serum (FBS, 50115, Biochrom, Holliston, MA, USA). The cell concentration was 15 × 10^3^ cells/well for the lung fibroblast cells and 2 × 10^4^ (Molm-13)/ 10^4^ (Molt-4) cells/well for the cancer cells. After seeding, the cells were incubated for 24 h at 37°C and 5% CO2. The medium was then replaced with fresh RPMI-1640 medium supplemented with 10% (v/v) FBS and gentamycin (10 μg/ml, A2712, Merck). After adding 10 μl of extract samples diluted in 2% (v/v) DMSO in pH_2_O, the cells were incubated for 72 h at 37°C and 5% CO2. For assaying the viability of the cells, 10 μl of CellTiter 96® AQueous One Solution reagent (G3581, Promega, Madison, WI, USA) containing tetrazolium [3-(4,5-dimethylthiazol-2-yl)-5-(3-carboxymethoxyphenyl)-2-(4-sulfophenyl)-2H-tetrazolium, inner salt] and phenazine ethosulfate were added to each well and incubated for 1 h. The assays were done with three technical replicates. The plates were read using a DTX 880 plate reader (Beckman Coulter, CA, USA) by measuring the absorbance at *λ* = 485 nm. The cell viability was calculated using the media control. As a negative control, RPMI-1640 with 10% (v/v) FBS and 10% (v/v) DMSO (Sigma) was used as a positive control.

### UHPLC-MS/MS analysis and identification of compounds

UHPLC-HR-MS data for dereplication was recorded using an Acquity I-class UPLC (Waters,Milford, MA, USA) coupled to a PDA detector and a Vion IMS QToF (Waters). The chromatographic separation was performed using an Acquity C-18 UPLC column (1.7 μm, 2.1 mm × 100 mm; Waters). Mobile phases consisted of acetonitrile (HiPerSolv, VWR) for mobile phase A and pH_2_O produced by the in-house Milli-Q system as mobile phase B, both containing 0.1% formic acid (v/v; 33015, Sigma). The gradient was run from 10 to 90% B over 12 min at a flow rate of 0.45 ml/min. Samples were run in ESI+ and ESI-ionization mode. The data was processed and analyzed using UNIFI 1.9.4 (Waters). Exact masses were calculated using ChemCalc (Patiny and Borel, 2013). For dereplication extracts of the respective cultivation media were prepared to exclude media components from consideration.

## Results

### Samples

**Figure 3:**
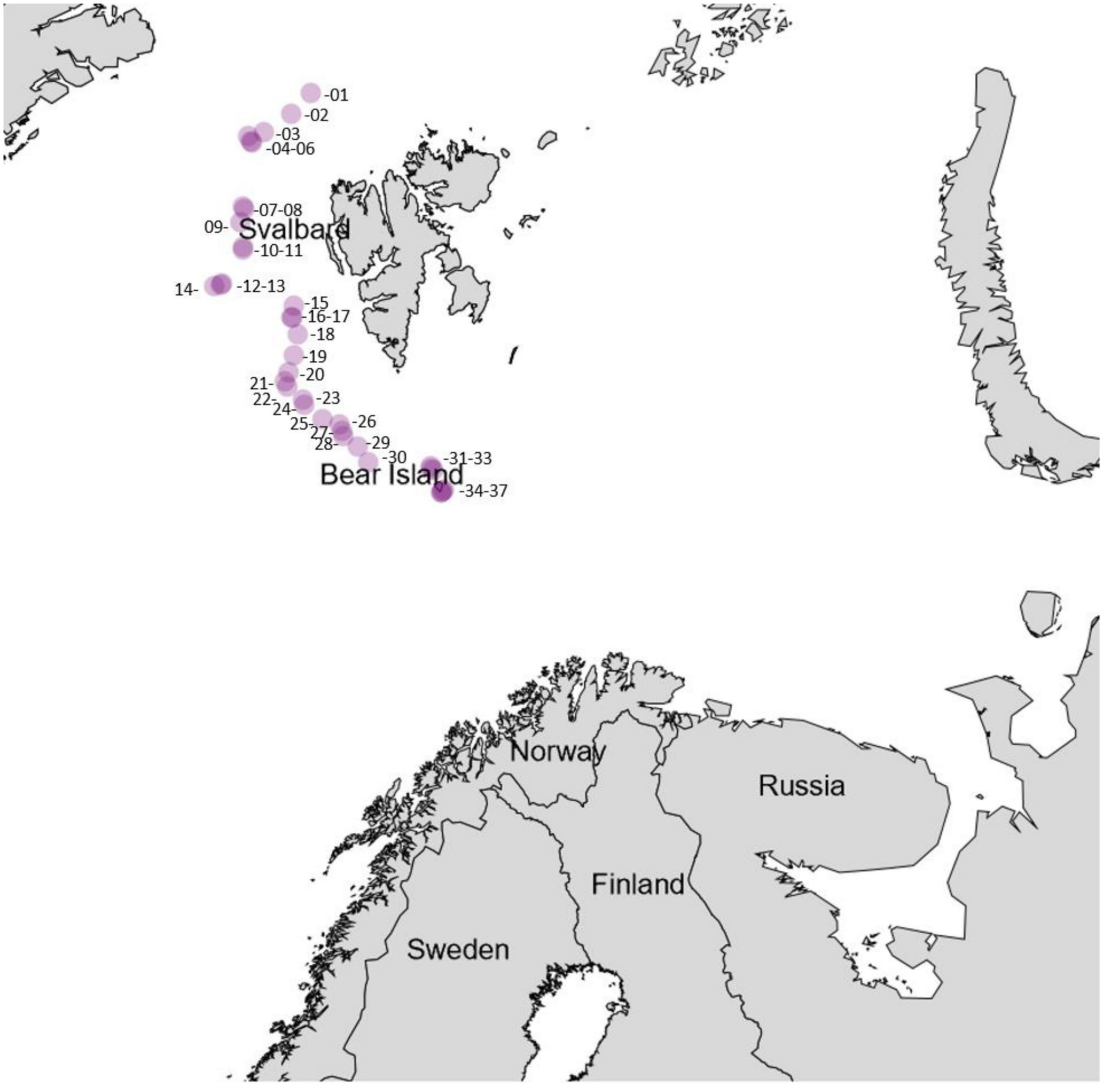
Sampling stations throughout the research cruise, the approximate coordinates are given in Supplementary Table 2 within the Supplementary information.

Samples for bacteria isolation were collected from 23 animals, 6 sediment cores, and one piece of driftwood collected by trawl during the cruise. Intertidal sediment and soil samples from Bjørnøya were sampled by hand. A map of the cruise track with the respective sampling sites is given in Figure 3. An exhaustive list of the coordinates of the sample sites is given in the Supplementary information. A list of the samples that were processed is given in Table 1.

**Table 1:** The list of the samples (including sediment, animals, drift-wood) and sampling sites (intertidal sediment and soil at Bjørnøya) from which actinobacteria have been isolated.

Animal-, and other samples collected			
Sample		Phylum (if applicable)	Sampling Site °N, °E (Sampling station, approx. Depth, Animal sample Nr.)
*Stylocordyla borealis*		Porifera	80.9508012, 5.571379683 (SST-02, 750 m, Animal 1)
*Asbestopluma* sp.		Porifera	80.33259517, 1.992459283 (SST-04, 2,200 m, Animal 4)
*Caulophacus arcticus*		Porifera	80.41478843, 1.614629867 (SST-05, 2,750 m, Animal 5)
*Thenea* sp.		Porifera	77.37491402, 8.268010617 (SST-17, 1,400 m, Animal 6)
*Geodia* sp.		Porifera	77.37491402, 8.268010617 (SST-17, 1,400 m, Animal 7)
*Geodia* sp.		Porifera	77.37491402, 8.268010617 (SST-17, 1,400 m, Animal 8)
*Caulophacus arcticus*		Porifera	77.37491402, 8.268010617 (SST-17, 1,400 m, Animal 10)
*Porifera* indet		Porifera	75.33318083, 12.71910378 (SST-28, 1800 m, Animal 17)
Presumably sponge remains		Presumably Porifera	80.9508012, 5.571379683 (SST-02, 750 m, Animal 2)
Presumably sponge remains		Presumably Porifera	80.9508012, 5.571379683 (SST-02, 750 m, Animal 3)
Presumably sponge remains		Presumably Porifera	75.86469082, 9.833769933 (SST-23, 2,300 m, Animal 11)
Presumably sponge remains		Presumably Porifera	75.86469082, 9.833769933 (SST-23, 2,300 m, Animal 12)
*Hormathia* sp.		Cnidaria	77.37491402, 8.268010617 (SST-17, 1,400 m, Animal 9)
*Thuiaria breitfussi*		Cnidaria	75.33318083, 12.71910378 (SST-28, 1800 m, Animal 15)
*Actiniaria* indet		Cnidaria	75.16057145, 13.71765962 (SST-29, 1,500 m, Animal 20)
*Halecium muricatum*		Cnidaria	75.16057145, 13.71765962 (SST-29, 1,500 m, Animal 21)
*Chlamys islandica*		Mollusca	74.78804448, 18.57096443 (SST-31, 285 m, Animal 23)
*Reteporella beaniana*		Bryozoa	75.86469082, 9.833769933 (SST-23, 2,300 m, Animal 13)
*Flustridae* indet		Bryozoa	75.95500417, 9.773894217 (SST-24, 2,200 m, Animal 14)
*Dendrobeania* sp.		Bryozoa	75.16057145, 13.71765962 (SST-29, 1,500 m, Animal 18)
*Tegella spitzbergensis*		Bryozoa	75.16057145, 13.71765962 (SST-29, 1,500 m, Animal 19)
*Tricellaria ternata*		Bryozoa	75.16057145, 13.71765962 (SST-29, 1,500 m, Animal 22)
*Synoicum turgens*		Chordata	75.33318083, 12.71910378 (SST-28, 1800 m, Animal 16)
Driftwood from bottom-trawl		-	79.13702795, 2.816979217 (SST-07, 5,600 m)
Sediment cores			
Nr. (ID)	Depth:		°N, °E (Sampling Site):
1	450 m		81.37719865, 7.49002125 (SST-01)
2	750 m		80.9508012, 5.571379683 (SST-02)
3	2,200 m		80.33259517, 1.992459283 (SST-04)
4	5,600 m		79.19013, 2.568842 (SST-08)
5	2,200 m		75.63154527, 11.23584792 (SST-25)
6	285 m		74.78804448, 18.57096443 (SST-31)
Sampling sites on Bjørnøya island			
Nr.	ID:		Approximate location, °N, °E:
1	Bjørnøya I		74.348479, 19.042081
2	Bjørnøya II		74.503094, 18.975873

### Bacterial isolates

**Figure 4:**
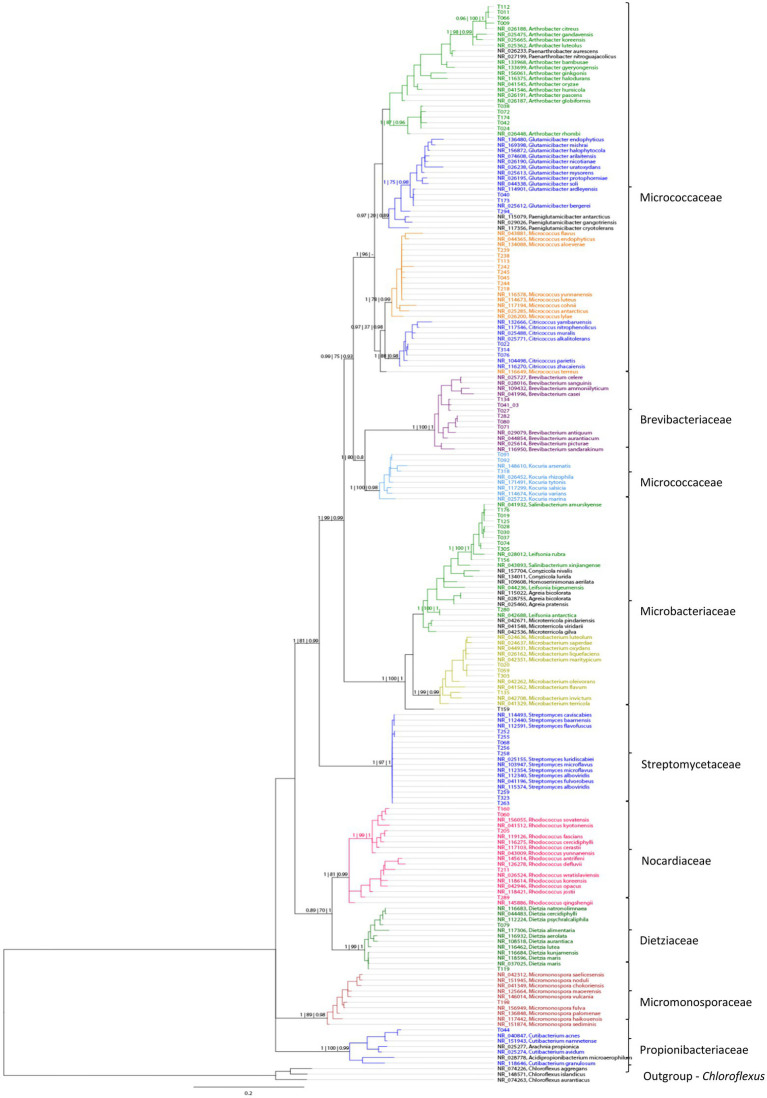
A phylogenetic tree based on comparison of 62 actinobacterial 16S rRNA sequences from 10 sampling sites, based on Bayesian analysis. New isolates are represented by numbers, while colors indicate their similarity to known taxa. Support values are given in the following order as Bayesian | Maximum likelihood | FastTree support.

**Figure 5:**
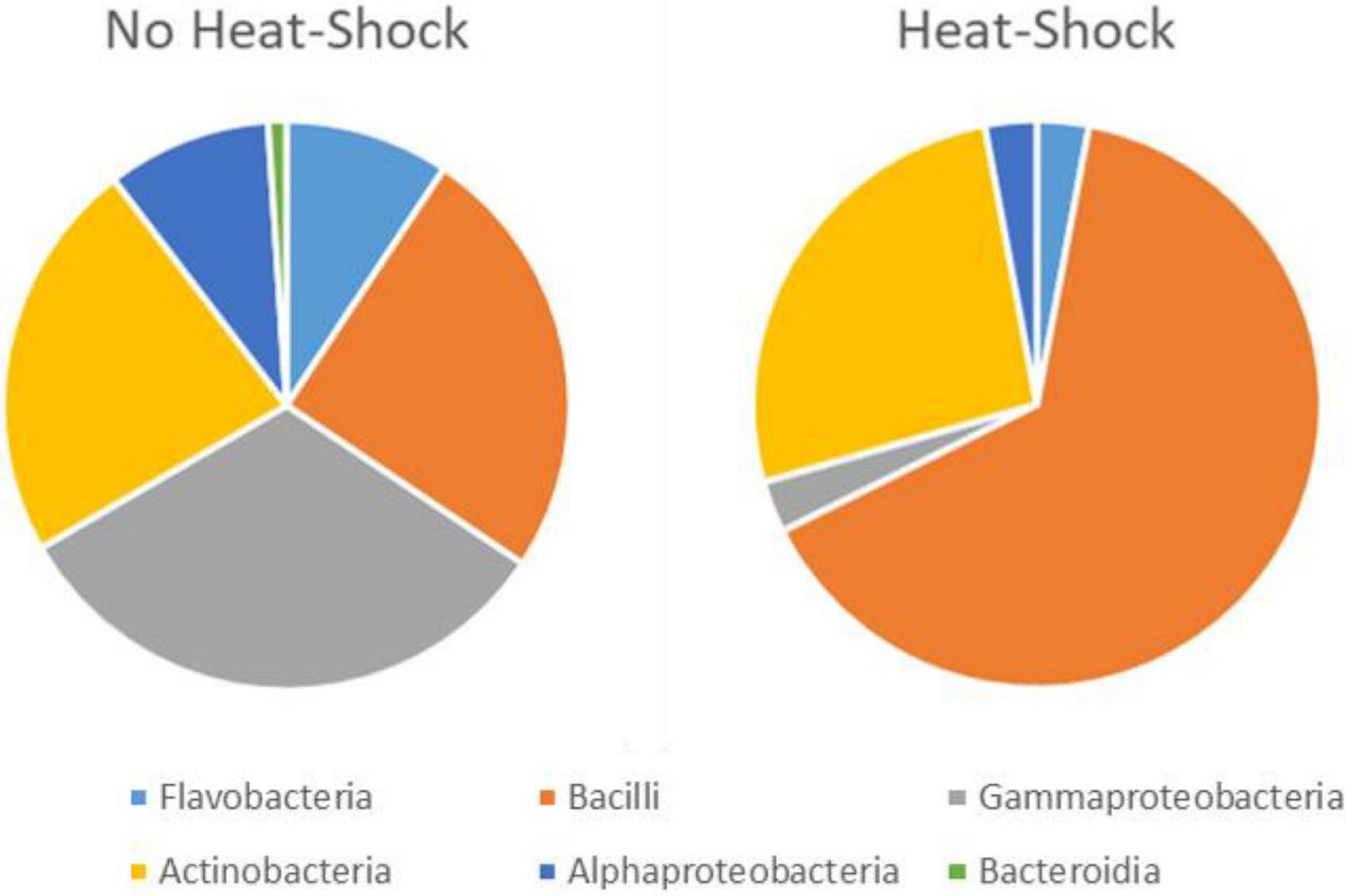
Effect of heat shock treatment on the isolation of spore forming bacteria. The total number of isolates is 68 after heat shock and 194 without heat shock. However, the quantitative representation of the bacterial groups varies significantly between heat shock and control.

**Figure 6:**
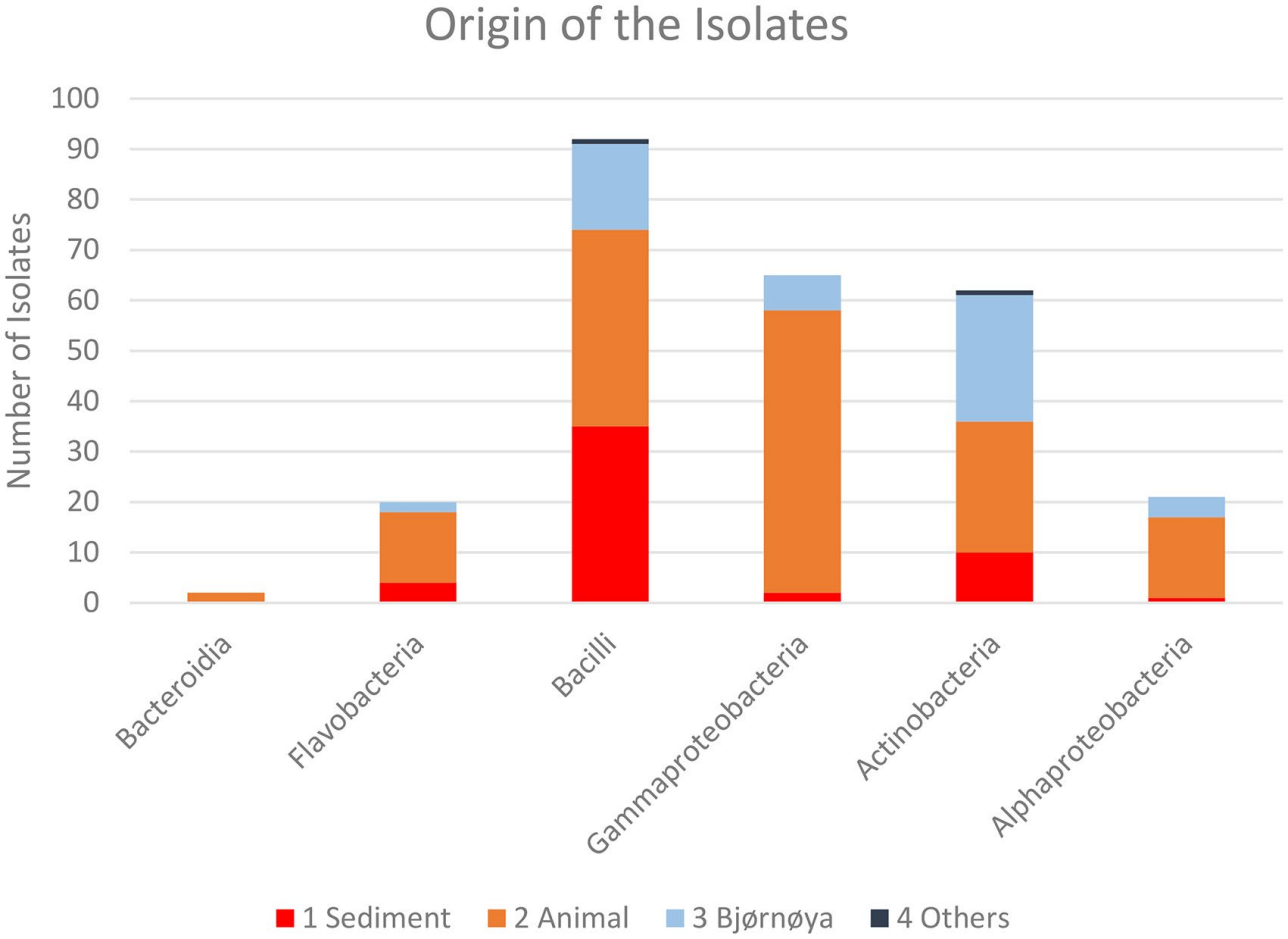
Taxonomic composition of the isolates recovered from the respective groups of sample sources for all sample treatments. Individual numbers and relative compositions are given in Table 3.

The number of isolates identified as “not actinobacteria” was registered for statistics but the isolates were not preserved. The 16S sequences used for identification of the actinobacteria isolates were deposited in gene bank (Gene bank IDs: OP537085 - OP537147). In total 262 isolates were obtained and of those 62 isolates were identified as actinobacteria. A list with sample origin, identity and sampling site can be found in the Supplementary information (Supplementary Table 1). A phylogenetic comparison of the isolates is given in Figure 4 and more detailed in Supplementary Figure 2. The 62 isolates were distributed among five orders, nine families and 15 genera. The most abundant isolates were *Leifsonia/Salinibacterium* (10), *Arthrobacter* (9), *Streptomyces* (8), and *Micrococcus* (8). The outgroup *Chloroflexus* was selected because it belongs to Chloroflexota which is the closest phylum to Actinomycetota according to Hug et al. (2016) For analyzing the efficacy of the different sample treatments, we grouped the isolates according to their sample-pretreatment and phylogenetic group (Figure 5), origin (animal, sediment etc. Figure 6 and Table 2), and isolation media (Table 3).

**Table 2:** Taxonomic composition of the isolates grouped among the tree isolation media, given are the numbers of respective isolates and relative numbers of isolates for the respective media in brackets.

	M1	R2A	AiA	Sum
Bacteroidia	2 (1.7)	0 (0.0)	0 (0.0)	2
Flavobacteria	8 (6.6)	6 (8.1)	6 (9.0)	20
Bacilli	53 (43.8)	25 (33.8)	14 (20.9)	92
Gammaproteobacteria	20 (16.5)	22 (29.7)	23 (34.3)	65
Actinobacteria	31 (25.6)	12 (16.2)	19 (28.4)	62
Alphaproteobacteria	7 (5.8)	9 (12.2)	5 (7.5)	21
Sum:	121	74	67	262

**Table 3:** Taxonomic composition of the isolates grouped among the four groups of sample types, given are the numbers of respective isolates and relative numbers of isolates for the respective media in brackets, a visualization is given in Figure 6.

	1 Sediment	2 Animal	3 Bjørnøya	4 Others	Sum
Bacteroidia	0 (0.0)	2 (1.3)	0 (0.0)	0 (0.0)	2
Flavobacteria	4 (7.7)	14 (9.2)	2 (3.6)	0 (0.0)	20
Bacilli	35 (67.3)	39 (25.5)	17 (30.9)	1 (50.0)*	92
Gammaproteobacteria	2 (3.8)	56 (36.6)	7 (12.7)	0 (0.0)	65
Actinobacteria	10 (19.2)	26 (17.0)	25 (45.5)	1 (50.0)*	62
Alphaproteobacteria	1 (1.9)	16 (10.5)	4 (7.3)	0 (0.0)	21
Sum:	52	153	55	2	262

* “Others” includes only two isolates, one bacillus and one actinobacteria isolated from driftwood.

For the heat treatment/ heat shock we can clearly see in Figure 5 that it significantly reduces the number of isolates obtained. The relative number of about 27% actinobacteria with heat-shock and about 23% without stays similar, however, the bacilli increased from 23 to 63% from the isolates when using the heat shock method. The number of other bacteria decreased from 54 to 10%. The numbers are given in Supplementary Table 3. In Table 2 the isolates obtained from different isolation media are grouped according to the taxonomic groups of isolates. Table 3 and Figure 6 show how many isolates from the different taxonomic groups where isolated from the different sample-classes. In Tables 2, 3 the relative numbers of isolates within a table-column are given in parenthesis.

**Figure 7:**
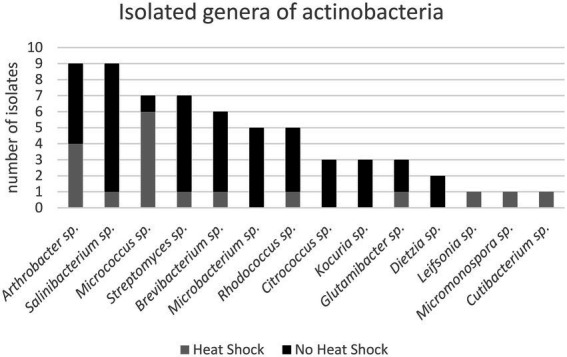
Isolated genera of actinobacteria according to tentative 16S rRNA sequencing and phylogenetic analysis.

In Figure 7 the different actinobacterial genera isolated and the respective number of isolates are given. We grouped the isolates according to the sample pre-treatment (heat-shock/ no heat-shock) in order to visualize a potential impact of the sample-pretreatment on the composition of actinobacteria isolates.

### Anti-microbial and anti-proliferative effects of the extracts

For screening the 62 actinobacterial isolates for anti-microbial activity, we tested a protocol using cultivation of the actinobacteria in 96-deep-well plates (3.0 ml vol./well) using a culture volume of 2.2 ml per isolate. Both, the supernatants and MeOH extracts of the cells were tested for anti-microbial activity. Using 100 μl of the culture-supernatant directly in the screening against the bacteria *Streptococcus agalactiae* and *Pseudomonas aeruginosa* showed increased growth, and no antimicrobial activity was detected. Using the MeOH extract for bioactivity testing we observed anti-microbial activity of three isolates (T036, T245 and T255) against *S. agalactiae* and no effect on *P. aeruginosa*.

To compare cultivation and extraction in deep-well-plates with our established resin extraction protocol and subsequent fractionation into six extract fraction using reversed-phase liquid-chromatography. We used the latter protocol for extract-production, fractionation and screening on four randomly selected actinobacteria-isolates. The bacteria were cultivated, extracted with a poly-benzyl-resin and fractionated using liquid chromatography. The bioactivity of the extract fractions were tested at concentrations of 100, 50, 25 and 10 μg/ml for anti-microbial effects against *Staphylococcus aureus*, *Escherichia coli*, *Enterococcus faecalis*, *Pseudomonas aeruginosa*, *Streptococcus agalactiae*, and Methicillin-resistant *Staphylococcus aureus* (MRSA) and anti-proliferative effects in two human cancer cell lines and one immortalized human lung-fibroblast cell-line. The results for the anti-bacterial testing are given in Tables 4, 5 show the results for the anti-proliferative-assays. The fractions that have shown bioactivity where further investigated for the presence of bioactive compounds using UHPLC–MS/MS, described under Section “Results of the metabolomic investigation.”

**Table 4:** Results of the anti-bacterial screening for the fractionated resin extracts.

Sample	Tested concentration:	
	100 μg/ml	50 μg/ml
T020 (*Microbacterium* sp.), fraction 05	*S. agal.; S. aur.*	*S. agal.*
T020 (*Microbacterium* sp.), fraction 06	*S. agal.; S. aur.*	–
T022 (*Citrococcus* sp.), all fractions	–	–
T045 (*Micrococcus* sp.), fraction 05	*S. agal.*	*S. agal.*
T045 (*Micrococcus* sp.), fraction 06	*S. agal.; S. aur.*	*S. agal.*
T252 (*Streptomyces* sp.), fraction 04	*S. aur.*	*S. aur.*
T252 (*Streptomyces* sp.), fraction 05	*S. agal.; S. aur.; E. faec.*	*S. agal.; S. aur.; E. faec.*

“–”: no bioactivity observed; all samples were tested in S. aureus, E. coli, E. faecialis, P. aeruginosa, S. agalactiae and MRSA. The bacteria on which anti-bacterial effect has been observed at the respective concentration of the extract-fraction is given in the table.

**Table 5:** Results of the anti-cancer screening for the fractionated resin extracts, given are the reduced cell proliferation in percent of the control (100%/ normal growth) for the respective test-concentration of extract fractions, which is given in parenthesis.

Sample	MV411	MOLT4	MRC5
T020 (*Microbacterium* sp.), fraction 05	35 (25 μg/ml)	58 (50 μg/ml)	35 (100 μg/ml)
T020 (*Microbacterium* sp.), fraction 06	35 (25 μg/ml)	4 (100 μg/ml)	–
T022 (*Citrococcus* sp.), all fractions	–	–	–
T045 (*Micrococcus* sp.), fraction 05	34 (50 μg/ml)	8 (100 μg/ml)	30 (100 μg/ml)
T045 (*Micrococcus* sp.), fraction 06	35 (25 μg/ml)	27 (50 μg/ml)	29 (100 μg/ml)
T252 (*Streptomyces* sp.), fraction 04	0 (25 μg/ml)	0 (25 μg/ml)	28 (10 μg/ml)
T252 (*Streptomyces* sp.), fraction 05	–	–	–

“–”: no bioactivity observed; all samples were tested at concentrations of 100, 50, 25 and 10 μg/ml. The lowest concentration of observed activity is given in parenthesis after the observed cell-proliferation in % (0% = total inhibition).

### Results of the metabolomic investigation

**Figure 8:**
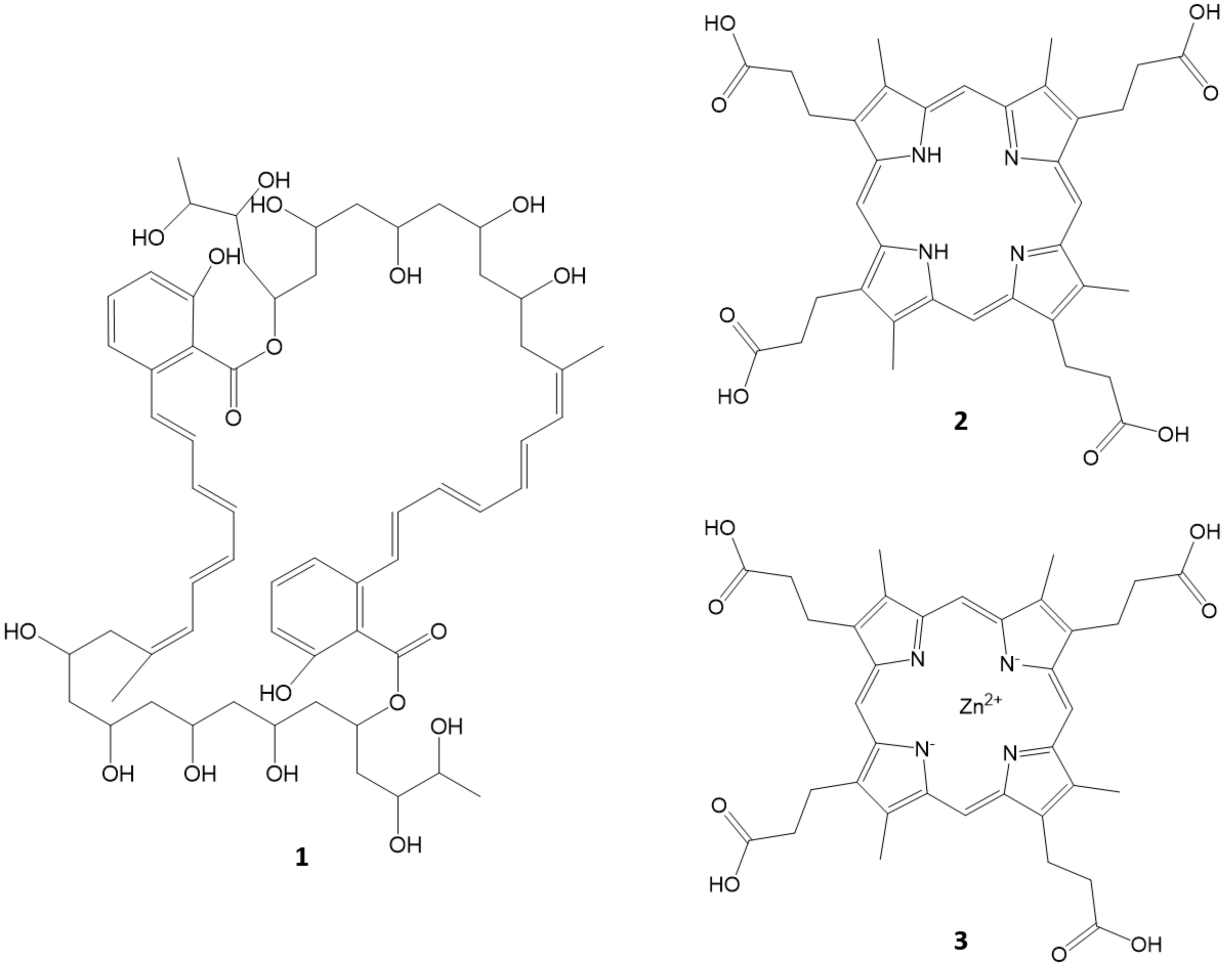
Compounds tentatively identified *via* MS/MS and UV/Vis spectra. SIA7248 (1), coproporphyrin III (2) and Zn coproporphyrin III (3).

A preliminary UHPLC–MS/MS analysis of the bacterial extracts that showed bioactivity in the assays tested revealed that the active FLASH fraction 05 of T252 contained a compound with *m/z* = 1093.57171 (C_60_H_84_O_18_, calcd. *m/z* = 1093.57359 [M + H]^+^), likely the marinomycin analogue SIA7248 (1; Zou et al., 2013). This could explain the anti-cancer and anti-bacterial effects of the fraction. The MS2 spectra as well as the UV/Vis spectra were in accordance with the published data (Zou et al., 2013), and they are given in the Supplementary information (Supplementary Figures 3–5). The compound eluted as several peaks between RT = 5.0 and 7.0 min (Supplementary Figure 3). However, SIA7248 could not be detected in the anti-bacterial fraction 04. The fractions of T022 did not show bioactivity but fractions 05 and 06 had a distinctive red color. Two compounds with the signals of *m/z* = 655.27579 (RT = 5.13 min) and *m/z* = 716.18188 (RT = 5.50 min) were detected, both showing strong absorption and characteristic UV/Vis spectra. The signal at of *m/z* = 655 was identified as [M-e]^+^ with a calculated elemental composition of C_36_H_39_N_4_O_8_ (calcd. *m/z* = 655.27679) literature research and inspection of the isotope satellites tentatively identified 716 as zinc coproporphyrin III (C_36_H_36_N_4_O_8_^64^Zn, calcd. *m/z* = 716.18246 [M-e]^+^). The two compounds are most likely coproporphyrin III and zink coproporphyrin III, and the UV/Vis and MS2 spectra can be found in the Supplementary information and comply with the published spectra (Cleary et al., 2018; Supplementary Figures 6, 7). An overview of the structures of identified compounds is given in Figure 8.

## Discussion

Several different sample pretreatment strategies can be applied to increase the ratio of actinobacterial isolates from the field samples. The choice of methods for isolation of bacteria in this project was somewhat restricted because we wanted to do the first step of the isolations onboard the research vessel, and there were certain research infrastructure limitations. Drying under laminar flow (Hameş-Kocabaş and Uzel, 2012) was not possible as there was no sterile laminar-flow bench available on board. Besides drying, chemicals such as phenol or benzethonium chloride in combination with a heat-treatment (Bredholt et al., 2008) or a single heat treatment (Hameş-Kocabaş and Uzel, 2012; Jiang et al., 2016) were possible alternatives described in the scientific literature. We decided to use the heat shock method (55°C for 10 min) which we could easily facilitate on-board using an electric thermo-block and sterile 1.5 ml reaction tubes. We compared the effect of heat shock to samples that were processed at ambient temperature. All isolation agar plates were supplemented with nalidixic acid and cycloheximide to prevent the growth of Gram negative bacteria and fungi, respectively. The number of plates manually processed during the isolation campaign was at least 414 plates, 150 isolation-agar plates and at least 262 FMAP agar plates since for some isolates more than one plating step was required in order to obtain axenic cultures the numbers are approximate.

Isolate identities were grouped in order to investigate the contributions of the sample pre-treatment, media or sample origin on the relative number of actinobacteria obtained. The first observation was the effect of the heat shock, visualized in Figure 5. The total number of isolates obtained was 196 without heat-shock and 68 when the samples were treated with heat shock. We conclude that the heat shock method (55°C for 10 min) is an efficient and easy way to reduce the number of non spore-forming bacterial isolates while increasing the relative yield of spore-forming bacteria including actinobacteria and bacilli. While the focus of this study was the isolation of marine actinobacteria, marine bacilli is also a group of bacteria, with higher biosynthetic potential (Schinke et al., 2017). Using the heat shock, we reduced the total number of bacteria and actinobacteria isolated, however, the share of actinobacteria was relatively constant while the share of bacilli increased significantly (see Figure 5). For our bioprospecting strategy which focuses on actinobacteria, it is desirable to find methods to reduce the relative number of bacilli in order to increase the relative share of actinobacteria per isolate.

For each sediment core one sample was taken from the recovered seabed surface and one sample from about-10 cm under seabed surface, the number of isolates differed. The surface samples yielded in total 43 isolates were 11 were actinobacteria in contrast to the sub-surface samples that only led to 8 isolates in total, seven bacilli and 1 actinobacteria. This indicates that the number of viable spores is significantly lower 10 cm under the seabed surface. Zaborska et al. reported an average sedimentation rate of 0.7 ± 0.4 mm p.a. for the western margin of the Barents Sea (Zaborska et al., 2008), that would indicate the sediment recovered from-10 cm is ~143 or between 91 and 333 years old.

The phylogenetical identities of the isolates from the different isolation media are given in Table 2 (relative numbers/ % of isolates per media in brackets). The same classifications for isolates are given in Figure 6 and Table 3 for the sample origin (animal, sediment, intertidal sediment and soil from Bjørnøya, and others). Comparing the results, it must be kept in mind that the sediment samples were plated solely on M1 media. An interesting observation is that the sediment gave (in relative numbers) about twice as many bacilli as the other sources. In relative yields of actinobacteria, the samples retrieved from Bjørnøya island yielded 45% actinobacteria, which includes intertidal and terrestrial sample-sites while for comparison the deep-sea sediment yielded 23.5% actinobacterial isolates. We obtained altogether 86% bacilli and actinobacteria isolates from the deep sea sediment samples and 14% of isolates belonging to “other” bacterial genera. Schinke et al. (2017) reviewed the anti-bacterial compounds isolated from marine bacteria from 2010 to 2015, and found that the majority the actinobacteria (around ¾) and bacilli (> ^1^/_2_) that produced anti-microbial compounds were isolated from sediments (see Schinke et al., 2017; Figure 2). These numbers can obviously be biased by differences in sampling efforts, since sediment samples are easily retrieved. The geographical location of the sample (near coast/ deep sea) apparently plays a role too, since water from terrestrial surface and rivers will transport terrestrial bacteria and spores (Bredholdt et al., 2007). It has been reported that the dominant groups of actinobacteria change with depth, from *Streptomyces* at the surface to more Micromonospora with increasing depth (Bredholdt et al., 2007). There are reports (Takahashi and Omura, 2003) that the abundance of actinobacteria is low in deep waters. The high abundance of actinobacteria in the terrestrial environment explains the high number of actinobacteria isolated from Bjørnøya sampling site I and II, both from soil and intertidal zone. Soil is generally known to be a fertile source of actinobacteria, which have also been successfully isolated from the intertidal zone (Jose and Jha, 2017).

Based on the observations of Schinke et al. (2017) we hypothesized that spores of spore-forming bacteria may sediment from the water column and thus making marine sediments a particular good source for spore-forming bacteria. The observation by Schinke et al. as well as our own observations support this when it comes to specificity of isolating spore-forming bacteria, not to a high total number of isolates.

Marine animals, such as sponges and other invertebrates have served as source for many actinobacterial isolates (Montalvo et al., 2005; Valliappan et al., 2014; Versluis et al., 2017). Thus, a high share of the sponge’s wet weight biomass can be symbiotic microorganisms (Hentschel et al., 2006; Santos-Gandelman et al., 2014) and the high yield of bacteria and actinobacteria from animals was therefore expected.

For the investigation of potential anti-microbial activity of the bacterial extracts we obtained extracts from the experimental 96-well plate down scale culture screening setup as well as from resin extraction from some of the bacterial isolates. For the down scaled cultures *P. aeruginosa* and *S. agalactiae* were used in order to investigate a potential effect on Gram positive and Gram negative bacteria since the quantity of extract was limited. *P. aeruginosa* represents a virulent Gram negative pathogen and is responsible for about 29% of the Gram negative bacterial infections in intensive care units (Vincent et al., 2009; Nathwani et al., 2014) and *S. agalactiae* was chosen as Gram positive test strain because in previous experiments it has been the most sensitive strain in our screening of anti-microbial compounds in extracts of bacterial cultures (Kristoffersen et al., 2018; Schneider et al., 2019, 2020). The two bacteria were used to test a proposed down-scaled culture screening protocol for isolates using cultivation and extraction in 96-deep-well plates. As a reference we used poly-benzylresin/liquid–solid phase extraction and subsequent biotesting for five randomly selected isolates, since the liquid–solid phase extraction is a laborious low-throughput technique. The extracts were fractionated into six fractions and subjected to biotesting for anti-bacterial and anti-proliferative activities. We have used liquid–solid phase extraction and fractionation successfully in earlier studies isolating natural products from marine bacteria and fungi (Schneider et al., 2019, 2020; Jenssen et al., 2021). We have also verified the capacity of our extraction protocol to isolate various natural products in a spiking study (Schneider et al., 2021). When using our down-scaled culture and a methanol extraction protocol four hits were found. Testing the culture supernatant directly in the anti-microbial assay did not result in any hits but on the contrary increased the growth of the assayed pathogens. We assume remaining media components and maybe nutrients released by dead bacteria in the supernatant have feed the pathogens. However, when using the five fractionated resin extracts, we observed three hits in the anti-bacterial screening. Resin extraction of bacterial fermentations is known to recover many media-components (Schneider et al., 2021). Fractionation of an extract has the potential to separate the compounds according to polarity and concentrate the active compound(s) up within one fraction (concentration of compound of interested relative to the matrix) and thereby overcome thresholds for bioactivity detection. However, we also discovered that the liquid–solid phase extraction was not suitable for the extraction of very polar compounds (Schneider et al., 2021) which is where methanol extraction or supernatant testing could theoretically have an edge. In the resin extraction we did not break up the bacterial cells as was done through freezing and methanol in the experimental protocol.

It is difficult to compare studies since many authors report only the obtained actinobacteria. The isolation methods and sample origins differ also greatly. In a recent study the major number of marine actinobacteria from a depth of 15 cm in northern Portugal were from the genus *Micromonospora*, a heat shock of 60°C for 5 min had an “minor” impact on the actinobacteria composition however, *Micromonospora*, followed by *Streptomyces* where the main genera. Some of our samples came from the intertidal zone of Bjørnøya, however we found only one *Micromonospora* isolate from deep-sea sediment (−5,600 m).

After detecting the bioactivities of some of the isolates we investigated the extracts and fractions for potentially known or unknown metabolites. We have tentatively identified cuproporphyrin III and zinc cuproporphyrin III in the extract of T022, the compound was isolated, investigated and has a very weak anti-fungal activity and is therefore not of further interest (Cleary et al., 2018). For T252 we could explain the anti-proliferative effect of fraction 05 upon cancer cells and bacteria by the tentatively identified polyketide SIA7248, an analogue of the anti-bacterial and anti-proliferative compound marinomycin (**4**, see Figure 7) isolated from the *Marinispora* genus of actinobacteria (Kwon et al., 2006). The compounds are identified with a high probability and since already known excluded from further investigation within the dereplication process (Cordell and Shin, 1999; Ito and Masubuchi, 2014; Gaudencio and Pereira, 2015). Further cultivation, extraction, bioactivity screening and chemical analysis will be executed in order to point out compounds for purification and structure elucidation that are more likely to be unknown.

## Conclusion

We conclude that heat shock is a simple and efficient method to increase the relative yield of spore and endospore forming isolates while reducing the total number of isolates when isolating marine actinobacteria from field samples. Relative to the total number of isolates, the deep-sea sediment samples yielded predominantly spore forming bacteria which we interpret as a consequence of spore sedimentation/enrichment within the sediment. From a quantitative point of view, the presence of bacteria in deeper sediment layers (−10 cm) decreases. A high number of actinobacteria relative to the total number of processed isolates is desirable when establishing a bacterial collection for the purpose of bioprospecting. After our investigation we conclude that the specific sampling of animals and deep-sea sediments in combination with heat-shock pre-treatment (using isolation agar supplemented with fungicides and antibiotics) is a suitable isolation protocol when targeting spore and endospore forming bacteria from the Arctic deep sea. Further studies could address the effectiveness of the antibiotics in the media but from our point of view more importantly, how to reduce the number of bacilli relative to the number of actinobacteria isolated.

## Data availability statement

The 16S sequences of the isolates were deposited in Gene bank (www.ncbi.nlm.nih.gov/genbank/) under the IDs: OP537085 - OP537147, further information on the isolates can be found within the Supplementary material.

## Author contributions

YS: study design, isolation of bacteria, extraction, metabolomics, bioactivity investigation, and manuscript writing. OH: identification of bacteria. CL and YS: molecular biology. EH and JA: cruise planning and execution, review, and finalization. All authors contributed to the article and approved the submitted version.

## Funding

The research cruise was funded by UiT-The Arctic University of Norway.

## Conflict of interest

The authors declare that the research was conducted in the absence of any commercial or financial relationships that could be construed as a potential conflict of interest.

## Publisher’s note

All claims expressed in this article are solely those of the authors and do not necessarily represent those of their affiliated organizations, or those of the publisher, the editors and the reviewers. Any product that may be evaluated in this article, or claim that may be made by its manufacturer, is not guaranteed or endorsed by the publisher.

## Footnotes

^1^ https://www.geneious.com
