# Supplementary material for: Selective isolation of Arctic marine actinobacteria and a down-scaled fermentation and extraction strategy for identifying bioactive compounds
Source: Front Microbiol. 2022 Nov 21;13:1005625. doi: 10.3389/fmicb.2022.1005625 (PMC9720112; doi:10.3389/fmicb.2022.1005625)
Supplement: Supplementary file 1 [file Data_Sheet_1.docx]

Supplementary Material

Selective isolation of Arctic marine Actinobacteria from field samples and a down-scaled fermentation and extraction strategy for identifying bioactive actinobacterial compounds.

Yannik K. Schneider^1*^, Ole C. Hagestad^2^, Chun Li^1^, Espen H. Hansen^1^, Jeanette H. Andersen^1^

^1^Marbio, Faculty for Fisheries, Biosciences and Economy, UiT—The Arctic University of Norway, Breivika, N-9037 Tromsø, Norway.

^2^Marbank, Institute of Marine Research, Breivika, N-9037 Tromsø, Norway.

* Correspondence:
Yannik K. Schneider
yannik.k.schneider@uit.no

# Supplementary Data

**Supplementary Table 1:** Table of isolated actinobacteria

**Supplementary Table 2:** Position and depth of the sampling stations

**Supplementary Figure 1:** Pictures of sampling and trawling equipment

**Supplementary Table 3:** Comparison of the isolates obtained after heat-shock

**Supplementary Figure 2:** Phylogenetic three of the isolated actinobacteria

**Supplementary Figure 3:** UV/Vis spectra of 1093

**Supplementary Figure 4:** Extracted ion chromatogram of 1093

**Supplementary Figure 5:** ESI-MS/MS spectra of 1093

**Supplementary Figure 6:** UV/Vis spectra of 655 and 716

**Supplementary Figure 7:** ESI-MS/MS spectra of 655 and 716

**Supplementary Table 1.** The list of isolated actinobacteria, origin (sample), sampling station (SST, coordinates for the sampling stations are given in supplementary Table 2), isolation media and genus level identification using16S rRNA sequencing, see also supplementary Figure 2 for the identification.

|  | **Isolate ID:** | **Origin:** | **Sampling site/ SST** | **Isolation Media** | **Heat shock** | **Genus level identification** |
| --- | --- | --- | --- | --- | --- | --- |
| 1 | T009 | sediment boxc. 1 surf. | SST-01 | M1 | yes | *Arthrobacter* sp. |
| 2 | T011 | soil surf. | Bjørnøya II | M1 | yes | *Arthrobacter* sp. |
| 3 | T019 | animal 17, sponge | SST-28 | M1 | no | *Salinibacterium* sp. |
| 4 | T020 | animal 17, sponge | SST-28 | M1 | no | *Microbacterium* sp. |
| 5 | T022 | animal 17, sponge | SST-28 | M1 | no | *Citrococcus* sp. |
| 6 | T024 | algae film on intertidal stones | Bjørnøya I | M1 | no | *Arthrobacter* sp. |
| 7 | T027 | intertidal sediment | Bjørnøya I | M1 | no | *Brevibacterium* sp. |
| 8 | T028 | intertidal sediment | Bjørnøya I | M1 | no | *Salinibacterium* sp. |
| 9 | T030 | algae film on intertidal stones | Bjørnøya I | R2A | no | *Salinibacterium* sp. |
| 10 | T037 | sediment, cave | Bjørnøya I | M1 | no | *Salinibacterium* sp. |
| 11 | T038 | sediment, cave | Bjørnøya I | M1 | no | *Arthrobacter* sp. |
| 12 | T040 | sediment, cave | Bjørnøya I | M1 | no | *Glutamibacter* sp. |
| 13 | T041-03 | sediment, cave | Bjørnøya I | M1 | no | *Brevibacterium* sp. |
| 14 | T042 | sediment (eutrophic) | Bjørnøya I | M1 | no | *Arthrobacter* sp. |
| 15 | T044 | sediment boxc. 1 surf. | SST-01 | M1 | yes | *Cutibacterium* sp. |
| 16 | T045 | sediment boxc. 1 surf. | SST-01 | M1 | yes | *Micrococcus* sp. |
| 17 | T048 | animal 17, sponge | SST-28 | R2A | no | *Microbacterium* sp. |
| 18 | T059 | animal 18, *Dendrobeania* sp. | SST-29 | M1 | no | *Microbacterium* sp. |
| 19 | T060 | animal 18, *Dendrobeania* sp. | SST-29 | M1 | no | *Rhodococcus* sp. |
| 20 | T066 | soil surf. | Bjørnøya II | AiA | yes | *Arthrobacter* sp. |
| 21 | T068 | soil surf. | Bjørnøya II | AiA | yes | *Streptomyces* sp. |
| 22 | T071 | algae film on intertidal stones | Bjørnøya I | AiA | no | *Brevibacterium* sp. |
| 23 | T072 | algae film on intertidal stones | Bjørnøya I | AiA | no | *Arthrobacter* sp. |
| 24 | T074 | algae film on intertidal stones | Bjørnøya I | AiA | no | *Salinibacterium* sp. |
| 25 | T076 | animal 18, *Dendrobeania* sp. | SST-29 | AiA | no | *Citrococcus* sp. |
| 26 | T079 | animal 16, *Synoicum turgens* | SST-28 | AiA | no | *Dietzia* sp. |
| 27 | T080 | animal 16, *Synoicum turgens* | SST-28 | AiA | no | *Brevibacterium* sp. |
| 28 | T091 | animal 10, *Caulophacus arcticus* | SST-17 | R2A | no | *Kocuria* sp. |
| 29 | T092 | animal 10, *Caulophacus arcticus* | SST-17 | R2A | no | *Kocuria* sp. |
| 30 | T112-01 | sediment boxc. 2 surf. | SST-02 | M1 | yes | *Arthrobacter* sp. |
| 31 | T113 | sediment boxc. 1 -10 cm | SST-01 | M1 | no | *Micrococcus* sp. |
| 32 | T119-01 | animal 18, *Dendrobeania* sp. | SST-29 | R2A | no | *Dietzia* sp. |
| 33 | T125 | animal 18, *Dendrobeania* sp. | SST-29 | R2A | no | *Salinibacterium* sp. |
| 34 | T134-02 | animal 19, *Tegella spitzbergensis* | SST-29 | M1 | no | *Brevibacterium* sp. |
| 35 | T135 | animal 19, *Tegella spitzbergensis* | SST-29 | M1 | no | *Microbacterium* sp. |
| 36 | T156 | sediment boxc. 2 surf. | SST-02 | M1 | no | *Salinibacterium* sp. |
| 37 | T159 | animal 22, *Tricellaria ternata* | SST-29 | R2A | no | *Microbacterium* sp. |
| 38 | T160-02 | animal 22, *Tricellaria ternata* | SST-29 | R2A | no | *Rhodococcus* sp. |
| 39 | T173 | animal 16, *Synoicum turgens* | SST-28 | M1 | no | *Glutamibacter* sp. |
| 40 | T174 | animal 16, *Synoicum turgens* | SST-28 | M1 | no | *Arthrobacter* sp. |
| 41 | T176-02 | animal 16, *Synoicum turgens* | SST-28 | M1 | no | *Salinibacterium* sp. |
| 42 | T198 | sediment boxc. 4 surf. | SST-08 | M1 | yes | *Micromonospora* sp. |
| 43 | T205 | algae film on intertidal stones | Bjørnøya I | R2A | yes | *Rhodococcus* sp. |
| 44 | T211 | sediment boxc. 4 surf. | SST-08 | M1 | no | *Rhodococcus* sp. |
| 45 | T218 | sediment boxc. 1 -10 cm | SST-01 | M1 | yes | *Micrococcus* sp. |
| 46 | T238 | driftwood | SST-07 | R2A | yes | *Micrococcus* sp. |
| 47 | T242 | animal 16, *Synoicum turgens* | SST-28 | R2A | yes | *Micrococcus* sp. |
| 48 | T244 | animal 3, sponge remains | SST-02 | AiA | yes | *Micrococcus* sp. |
| 49 | T245 | animal 1, *Stylochordyla borealis* | SST-02 | AiA | yes | *Micrococcus* sp. |
| 50 | T252 | soil | Bjørnøya II | AiA | no | *Streptomyces* sp. |
| 51 | T255 | soil | Bjørnøya II | AiA | no | *Streptomyces* sp. |
| 52 | T256 | soil | Bjørnøya II | AiA | no | *Streptomyces* sp. |
| 53 | T259 | soil | Bjørnøya II | AiA | no | *Streptomyces* sp. |
| 54 | T263 | soil | Bjørnøya II | AiA | no | *Streptomyces* sp. |
| 55 | T280 | sediment (eutrophic) | Bjørnøya I | M1 | yes | *Leifsonia* sp. |
| 56 | T282 | algae film on intertidal stones | Bjørnøya I | M1 | yes | *Brevibacterium* sp. |
| 57 | T289 | animal 23, *Chlamys islandica* | SST-31 | R2A | no | *Rhodococcus* sp. |
| 58 | T294 | sediment boxc. 4 surface | SST-08 | M1 | yes | *Glutamibacter* sp. |
| 59 | T303 | animal 21, *Halecium muricatum* | SST-29 | R2A | no | *Microbacterium* sp. |
| 60 | T305 | algae film on intertidal stones | Bjørnøya I | AiA | yes | *Salinibacterium* sp. |
| 61 | T314 | animal 17, sponge | SST28 | AiA | no | *Citrococcus* sp. |
| 62 | T318 | animal 22, *Tricellaria ternata* | SST-29 | AiA | no | *Kocuria* sp. |
| 63 | T323 | soil | Bjørnøya II | AiA | no | *Streptomyces* sp. |

**Supplementary Table 2:** Position and depth of the sampling stations (SST).

| **SST Nr.:** | **Latitude °N** | **Longitude** **°E** | **approx. depth [m]** |
| --- | --- | --- | --- |
| 1 | 81.37719865 | 7.49002125 | 450 |
| 2 | 80.9508012 | 5.571379683 | 750 |
| 3 | 80.534776 | 3.043290067 | 1200 |
| 4 | 80.33259517 | 1.992459283 | 2200 |
| 5 | 80.41478843 | 1.614629867 | 2750 |
| 6 | 80.31531762 | 2.0831879 | 2000 |
| 7 | 79.13702795 | 2.816979217 | 5600 |
| 8 | 79.1901285 | 2.5688416 | 5600 |
| 9 | 78.89946627 | 2.645863417 | 2420 |
| 10 | 78.47684405 | 3.40345175 | 2400 |
| 11 | 78.42585288 | 3.3889151 | 3000 |
| 12 | 77.78491003 | 2.2925986 | 3300 |
| 13 | 77.74358225 | 2.24645315 | 3200 |
| 14 | 77.70770408 | 1.738933467 | 3200 |
| 15 | 77.591832 | 8.311453267 | 2200 |
| 16 | 77.36985593 | 8.283278517 | 1400 |
| 17 | 77.37491402 | 8.268010617 | 1400 |
| 18 | 77.07979728 | 8.863405883 | 2200 |
| 19 | 76.70149475 | 8.773346433 | 2200 |
| 20 | 76.40822167 | 8.499455167 | 2300 |
| 21 | 76.2278157 | 8.30366855 | 2000 |
| 22 | 76.15589453 | 8.496510833 | 2200 |
| 23 | 75.86469082 | 9.833769933 | 2300 |
| 24 | 75.95500417 | 9.773894217 | 2200 |
| 25 | 75.63154527 | 11.23584792 | 2200 |
| 26 | 75.54639232 | 12.45679845 | 1700 |
| 27 | 75.43599273 | 12.66216748 | 1700 |
| 28 | 75.33318083 | 12.71910378 | 1800 |
| 29 | 75.16057145 | 13.71765962 | 1500 |
| 30 | 74.88997632 | 14.44824272 | 1635 |
| 31 | 74.78804448 | 18.57096443 | 285 |
| 32 | 74.76036917 | 18.60796183 | 180 |
| 33 | 74.7163649 | 18.7070802 | 138 |
| 34 | 74.3287726 | 19.13150027 | 40 |
| 35 | 74.33530603 | 19.15451168 | 40 |
| 36 | 74.37198507 | 19.30358707 | 40 |
| 37 | 74.34735727 | 19.23358603 | 40 |


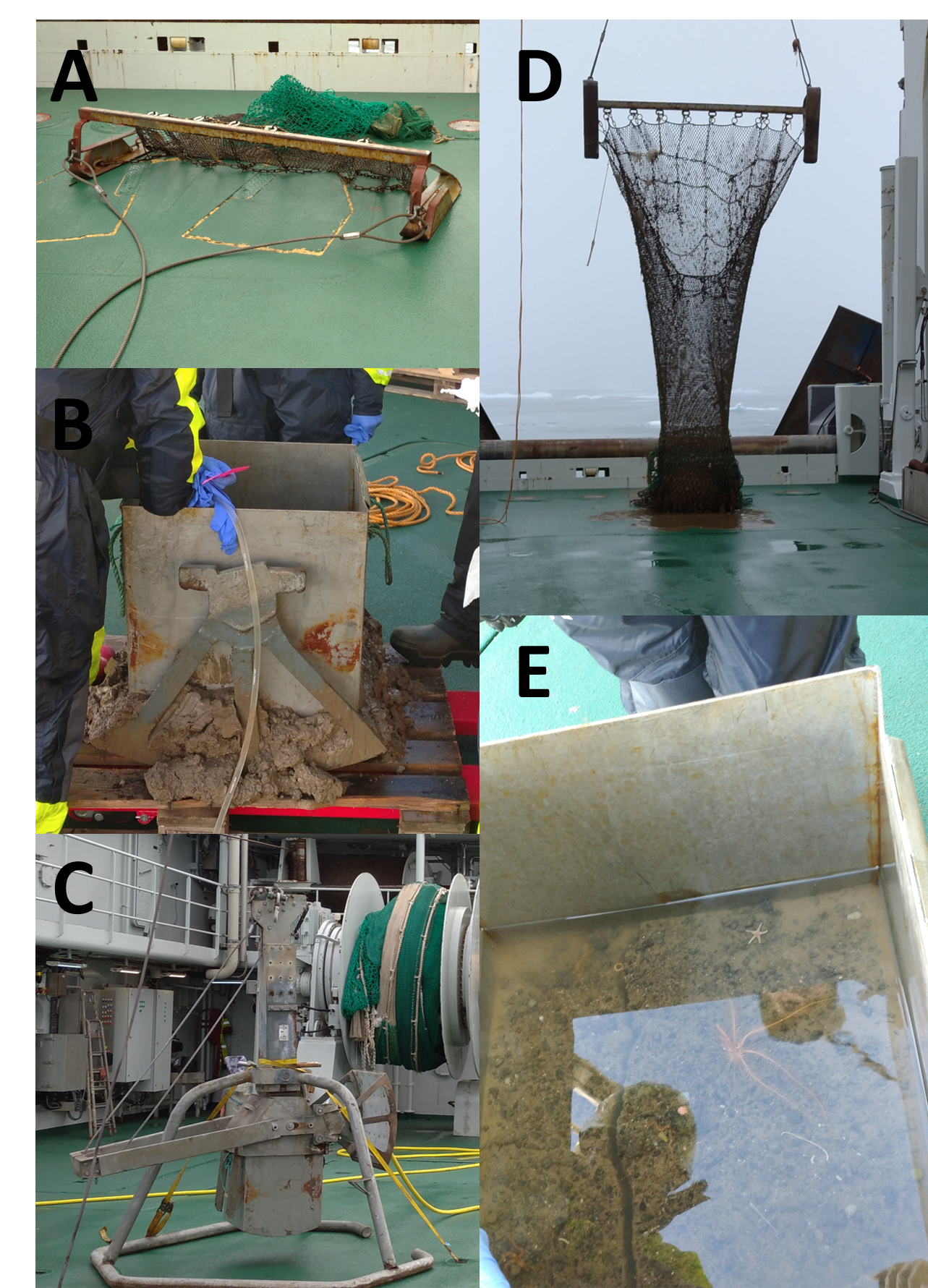


**Supplementary Figure 1.** Pictures of sampling and trawling equipment. **A**: Beam trawl for bottom trawling; **B**: Retrieved sediment box-core; **C**: box corer; **D**: Heave aboard of a beam trawl; **E**: Surface of a retrieved sediment box core. © Yannik Schneider.

**Supplementary Table 3:** Comparison of the isolates obtained after heat shock treatment of the samples.

|  | no Heat shock | Heat shock |
| --- | --- | --- |
| Bacteroidia | 2 | 0 |
| Flavobacteria | 18 | 2 |
| Bacilli | 48 | 44 |
| Gammaproteobacteria | 62 | 2 |
| Actinobacteria | 44 | 18 |
| Alphaproteobacteria | 18 | 2 |
| Sum: | 192 | 68 |


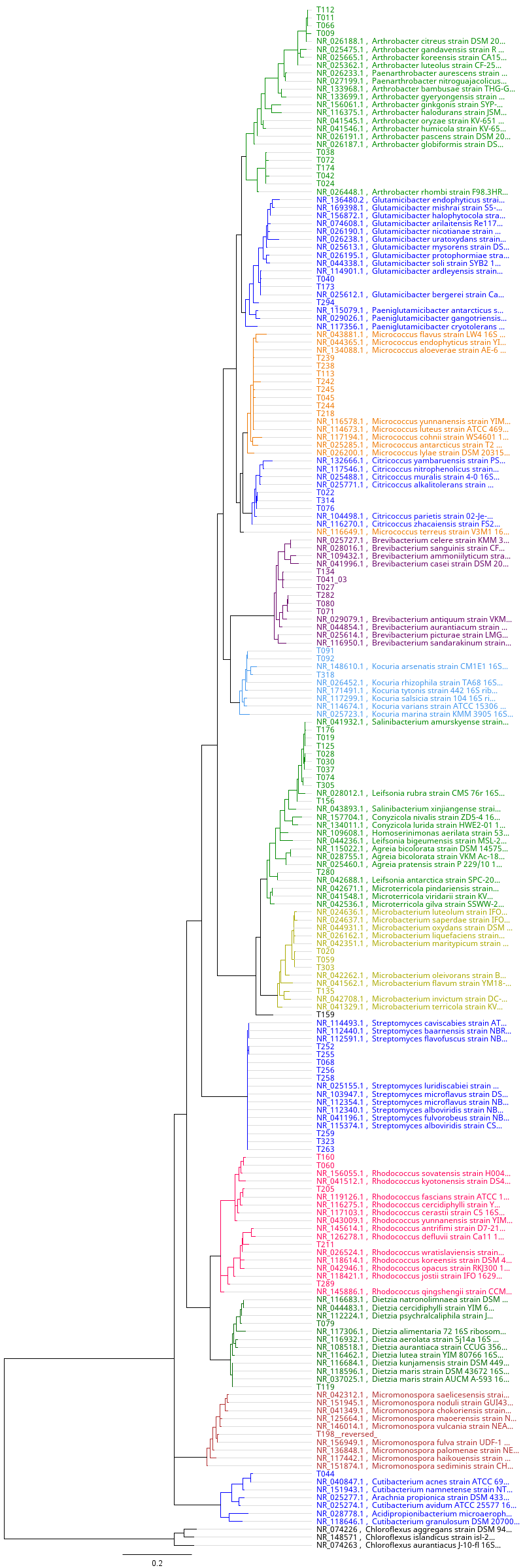

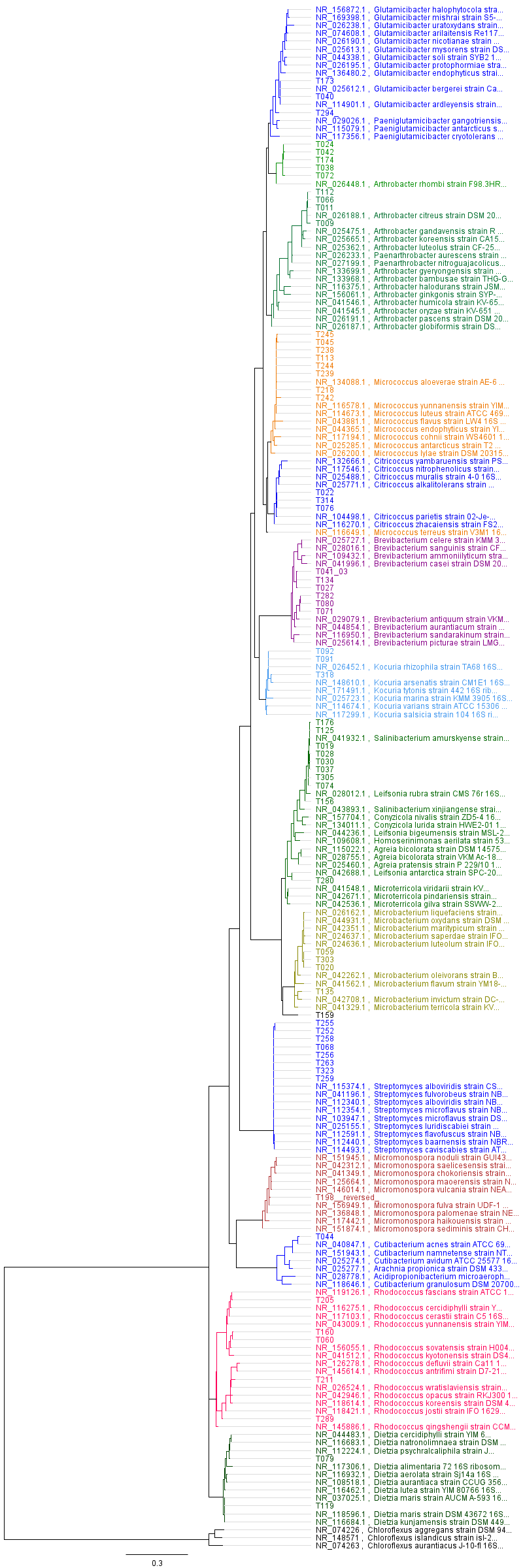


***Arthrobacter***

***Glutamibacter


Micrococcus


Citricoccus


Brevibacterium


Kocuria


Salinibacterium/Leifsonia


Microbacterium


Streptomyces


Rhodococcus


Dietzia


Micromonospora
Cutibacterium*
Outgroup - *Chloroflexus***

**B**

**A**

**Supplementary Figure 2.** A phylogenetic tree based on comparison of 62 actinobacterial 16S rRNA sequences from 10 sampling sites. Support values are given in the following order as Bayesian (A)| Maximum likelihood Bootstrap support | Fasttree support (B).


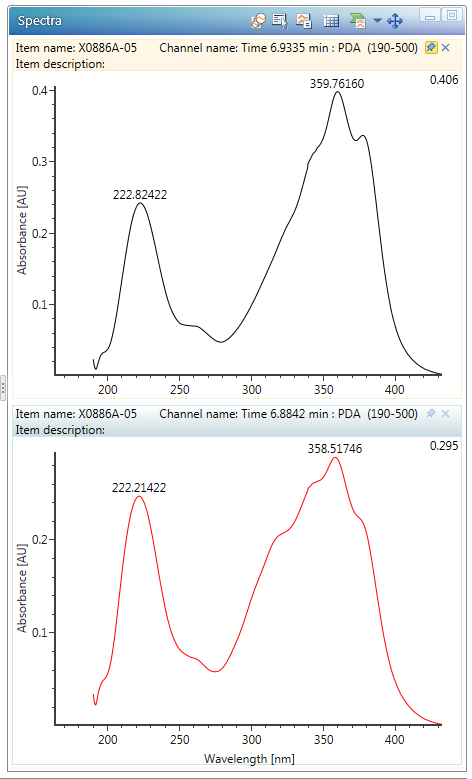


**Supplementary Figure 3.** UV/Vis spectra of 1093, taken in H_2_O:ACN.


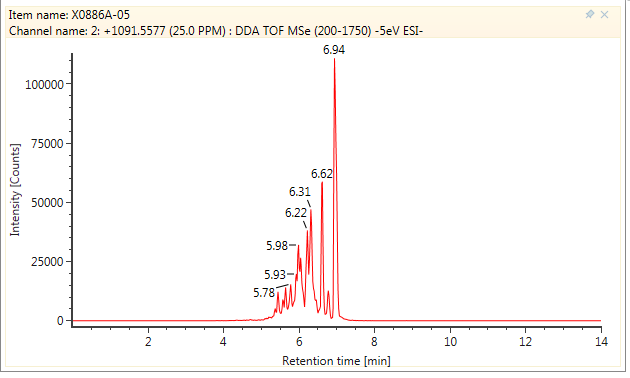


**Supplementary Figure 4.** Extracted Ion chromatogram of *m/z* = 1091.5577 ± 25.0 ppm.


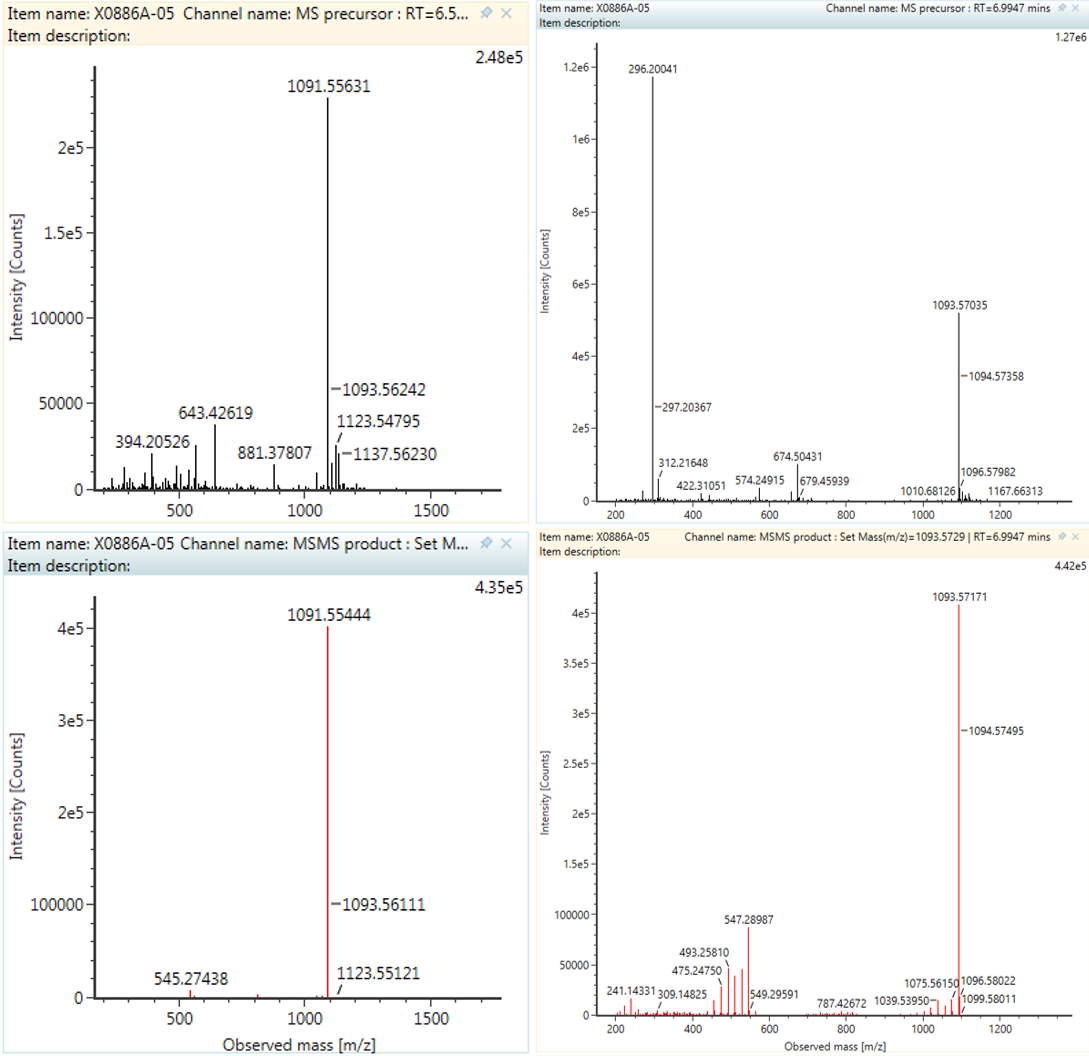


**Supplementary Figure 5.** ESI-MS/MS spectra of the compound 1093. To the right are the ESI- spectra and to the right are the ESI+ spectra. The low energy collision spectra are given in black and the high energy collision spectra are given in red.


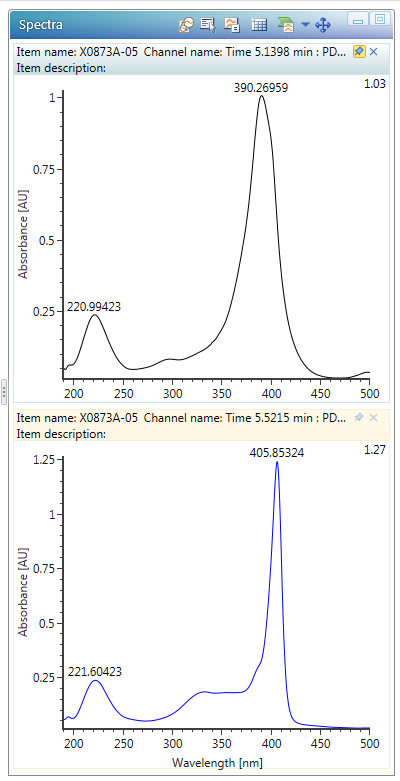


**Supplementary Figure 6.** UV/Vis spectra of 655 (black/top) and 716 (blue/bottom), both taken in H_2_O:ACN.


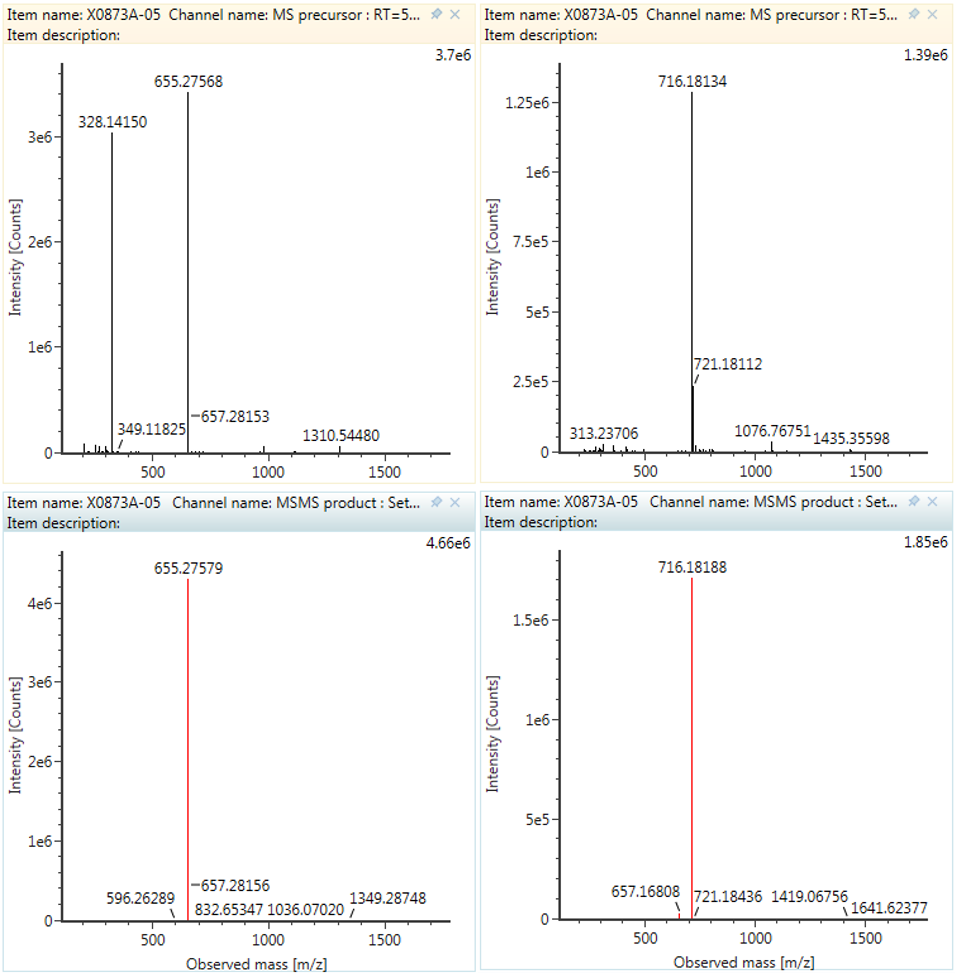


**Supplementary Figure 7.** MS/MS spectra of 655 (left) and 716 (right), the precursor ion spectra are given at the top/ in black and the product ion for the respective (60eV collision energy ramp).
